# Fifth Strategic Plan for the Development of Epidemiology in Brazil (2025–2029)

**DOI:** 10.1590/1980-549720250001.supl.1

**Published:** 2025-11-17

**Authors:** Tânia Maria de Araújo, Enirtes Caetano Prates Melo, Claudia Leite de Moraes, Laio Magno, Ana Paula Muraro, Maria de Jesus Mendes da Fonseca, Alberto Novaes Ramos, Luana Giatti Gonçalves, Margareth Guimarães Lima, Ligia Regina de Oliveira, Moisés Goldbaum, Joilda Silva Nery, Maria Rita Donalisio, Maria da Glória Lima Cruz Teixeira, Katia Vergetti Bloch, Antonio Fernando Boing, Guilherme Loureiro Werneck, *Strategic Plan Steering Committee, Thematic Area I: Training in Epidemiology, Amanda de Moura Souza, Edna Massae Yokoo, Elisabeth Carmen Duarte, Emanuele Souza Marques, Janaína dos Santos Motta, João Roberto Cavalcante Sampaio, Juan José Cortez Escalante, Katia Crestine Poças, Lívia Teixeira Souza Maia, Michael Eduardo Reichenheim, Naomar Monteiro de Almeida-Filho, Paola Barbosa Marchesini, Rodrigo Citton Padilha dos Reis, Sérgio Viana Peixoto, Andréia Moreira de Andrade, Antônio Augusto Moura da Silva, Carolina Geralda Alves, Felipe Guimarães Tavares, Fernando José Herkrath, Isis Polianna Silva Ferreira de Carvalho, Jesem Douglas Yamall Orellana, Ligia Regina Franco Sansigolo Kerr, Maria Amélia de Sousa Mascena Veras, Maria Cynthia Braga, Maria do Carmo Leal, Marilisa Berti de Azevedo Barros, Thematic Area II: Research in Epidemiology, Ana Paula Nogueira Nunes, Mauricio Lima Barreto, Paloma de Sousa Pinho, Paulo Rossi Menezes, Sandhi Maria Barreto, Vinícius Silva Belo, Wildo Navegantes de Araújo, Ana Paula Nogueira Nunes, Andréia Moreira de Andrade, Antônio Augusto Moura da Silva, Carolina Geralda Alves, Felipe Guimarães Tavares, Fernando José Herkrath, Isis Polianna Silva Ferreira de Carvalho, Jesem Douglas Yamall Orellana, Ligia Regina Franco Sansigolo Kerr, Maria Amélia de Sousa Mascena Veras, Maria Cynthia Braga, Maria do Carmo Leal, Marilisa Berti de Azevedo Barros, Mauricio Lima Barreto, Paloma de Sousa Pinho, Paulo Rossi Menezes, Sandhi Maria Barreto, Vinícius Silva Belo, Wildo Navegantes de Araújo, Thematic Area: Epidemiology in Policies, Programs, and Health Services, Alícia Krüger, Amarílis Bahia Bezerra, Bárbara Campos Silva Valente, Carmen Silvia Bruniera Domingues, Claudio Maierovitch Pessanha Henriques, Ethel Leonor Noia Maciel, Fatima Sonally Sousa Gondim, Gerusa Belo Gibson dos Santos, Gulnar Azevedo e Silva, Gustavo Laine Araújo de Oliveira, Heleno Rodrigues Corrêa, Luciana de Carvalho Penna, Maria Juliana Moura Corrêa, Marli Souza Rocha, Maryane Oliveira Campos, Olavo de Moura Fontoura, Sheila Maria Alvim de Matos, Walter Massa Ramalho, Alícia Krüger, Amarílis Bahia Bezerra, Bárbara Campos Silva Valente, Carmen Silvia Bruniera Domingues, Claudio Maierovitch Pessanha Henriques, Ethel Leonor Noia Maciel, Fatima Sonally Sousa Gondim, Gerusa Belo Gibson dos Santos, Gulnar Azevedo e Silva, Gustavo Laine Araújo de Oliveira, Heleno Rodrigues Corrêa, Luciana de Carvalho Penna, Maria Juliana Moura Corrêa, Marli Souza Rocha, Maryane Oliveira Campos, Olavo de Moura Fontoura, Sheila Maria Alvim de Matos, Walter Massa Ramalho

**Affiliations:** I Universidade Estadual de Feira de Santana, Epidemiology Center –, Feira de Santana (BA), Brazil.; II Fundação Oswaldo Cruz, National School of Public Health – Rio de Janeiro (RJ), Brazil.; III Universidade do Estado do Rio de Janeiro, Institute of Social Medicine – Rio de Janeiro (RJ), Brazil.; IV Universidade do Estado da Bahia, Department of Life Sciences – Salvador (BA), Brazil.; V Universidade Federal de Mato Grosso, Institute of Collective Health – Cuiabá (MT), Brazil.; VI Universidade Federal do Ceará, School of Medicine – Fortaleza (CE), Brazil.; VII Universidade Federal de Minas Gerais, School of Medicine – Belo Horizonte (MG), Brazil.; VIII Universidade Estadual de Campinas, School of Medical Sciences – Campinas (SP), Brazil.; IX Universidade de São Paulo, School of Medicine – São Paulo (SP), Brazil.; X Universidade Federal da Bahia, Institute of Collective Health – Salvador (BA), Brazil.; XI Universidade Federal do Rio de Janeiro, Institute for Collective Health Studies – Rio de Janeiro (RJ), Brazil.; XII Universidade Federal de Santa Catarina, Department of Public Health – Florianópolis (SC), Brazil.; XIII Ministério da Saúde, Secretariat of Health and Environmental Surveillance – Brasília (DF), Brazil.; Universidade Federal do Rio de Janeiro - Rio de Janeiro (RJ), Brazil.; Universidade Federal Fluminense - Niterói (RJ), Brazil.; Universidade de Brasília - Brasília (DF), Brazil.; Universidade do Estado do Rio de Janeiro - Rio de Janeiro (RJ), Brazil.; Universidade Federal de Pelotas - Pelotas (RS), Brazil.; Ministry of Health, Secretariat of Health and Environmental Surveillance - Brasília (DF), Brazil.; Pan American Health Organization - Brasília (DF), Brazil.; World Health Organization - Brasília (DF), Brazil.; Universidade de Brasília - Brasília (DF), Brazil.; Universidade Federal de Pernambuco - Vitória de Santo Antão (PE), Brazil.; Universidade do Estado do Rio de Janeiro - Rio de Janeiro (RJ), Brazil.; Universidade Federal da Bahia - Salvador (BA), Brazil.; Ministry of Health, Secretariat of Health and Environmental Surveillance - Brasília (DF), Brazil.; Universidade Federal do Rio Grande do Sul - Porto Alegre (RS), Brazil.; Fundação Oswaldo Cruz - Belo Horizonte (MG), Brazil.; Universidade Federal dos Vales do Jequitinhonha e Mucuri - Diamantina (MG), Brazil.; Universidade Federal do Acre - Rio Branco (AC), Brazil.; Universidade Federal do Maranhão - São Luís (MA), Brazil.; Ministry of Health, Secretariat of Health and Environmental Surveillance - Brasília (DF), Brazil.; Universidade Federal Fluminense - Niterói (RJ), Brazil.; Fundação Oswaldo Cruz - Manaus (AM), Brazil.; Ministry of Health, Secretariat of Health and Environmental Surveillance - Brasília (DF), Brazil.; Fundação Oswaldo Cruz - Manaus (AM), Brazil.; Universidade Federal do Ceará - Fortaleza (CE), Brazil.; Faculdade de Ciências Médicas da Santa Casa de São Paulo - São Paulo (SP), Brazil.; Fundação Oswaldo Cruz - Recife (PE), Brazil.; Fundação Oswaldo Cruz - Rio de Janeiro (RJ), Brazil.; Universidade Estadual de Campinas - Campinas (SP), Brazil.; Universidade Federal da Bahia - Salvador (BA), Brazil.; Universidade Federal do Recôncavo da Bahia - Santo Antônio de Jesus (BA), Brazil.; Universidade de São Paulo - São Paulo (SP), Brazil.; Universidade Federal de Minas Gerais - Belo Horizonte (MG), Brazil.; Universidade Federal de São João Del-Rei - Divinópolis (MG), Brazil.; Universidade de Brasília - Brasília (DF), Brazil.; Ministry of Health, Secretariat of Health and Environmental Surveillance - Brasília (DF), Brazil.; Pan American Health Organization - Brasília (DF), Brazil.; Fundação Oswaldo Cruz - Rio de Janeiro (RJ), Brazil.; Secretaria de Estado da Saúde de São Paulo - São Paulo (SP), Brazil.; Fundação Oswaldo Cruz - Brasília (DF), Brazil.; Ministry of Health, Secretariat of Health and Environmental Surveillance - Brasília (DF), Brazil.; Universidade Federal do Espírito Santo - Vitória (ES), Brazil.; Ministry of Health, Secretariat of Health and Environmental Surveillance - Brasília (DF), Brazil.; Universidade Federal do Rio de Janeiro - Rio de Janeiro (RJ), Brazil.; Universidade Estadual do Rio de Janeiro - Rio de Janeiro (RJ), Brazil.; Ministry of Health, Secretariat of Health and Environmental Surveillance - Brasília (DF), Brazil.; Universidade de Brasília - Brasília (DF), Brazil.; Ministry of Health, Secretariat of Health and Environmental Surveillance - Brasília (DF), Brazil.; Ministry of Health, Secretariat of Health and Environmental Surveillance - Brasília (DF), Brazil.; Ministry of Health, Secretariat of Health and Environmental Surveillance - Brasília (DF), Brazil.; Ministry of Health, Secretariat of Health and Environmental Surveillance - Brasília (DF), Brazil.; Ministry of Health, Secretariat of Health and Environmental Surveillance - Brasília (DF), Brazil.; Universidade Federal da Bahia - Salvador (BA), Brazil.; Universidade de Brasília - Brasília (DF), Brazil.

**Keywords:** Epidemiology, Strategic planning, Health education, Research, Health policy

## Abstract

The Brazilian Association of Collective Health (Abrasco) has played a leading role in consolidating collective health in Brazil since its foundation. After 19 years, Abrasco's Epidemiology Committee resumed its planning with the development of the Fifth Strategic Plan for the Development of Epidemiology in Brazil (2025–2029). This plan emerged in a challenging context marked by social inequalities, scientific denialism, the COVID-19 pandemic, the climate crisis, and disinvestment in public policies, reinforcing the need to update Brazilian epidemiology. The plan's development occurred in multiple stages, including online surveys, debates, and in-person workshops. Workshop I (Brasília, 2023) identified priority challenges, and Workshop II (Rio de Janeiro, 2024) defined strategies to address them. The construction process was guided by the qualified participation of epidemiologists from all regions of the country and was based on principles of plurality, diversity, gender equity, and race/ethnicity. The plan highlights epidemiology across three thematic areas: education, research, and health policies, programs, and services. The Strategic Plan Working Group of the Epidemiology Committee, supported by the Secretariat of Health and Environment Surveillance of the Ministry of Health, led the process. This supplement of the *Brazilian Journal of Epidemiology* presents the Fifth Strategic Plan for the Development of Epidemiology in Brazil (2025–2029), contributions to the discussion of some central topics, and a deepening of strategic and operational dimensions. The Fifth Strategic Plan for the Development of Epidemiology in Brazil (2025–2029) presents a collective vision for the future, aiming to strengthen epidemiology as a central discipline within the field of collective health. It reaffirms its commitment to equity, social justice, and the consolidation of the Sistema Único de Saúde in Brazil.

## PRESENTATION

Forty-five years ago, the Brazilian Association of Public Health (*Associação Brasileira de Saúde Coletiva* – Abrasco) — then called the Brazilian Association of Postgraduate Studies in Public Health — was established. Created by prominent activists in public health and in social and preventive medicine, Abrasco became a central forum in the struggle for health reform, while also aligning with social movements that demanded the democratization of the country^1^. During a period in which the Brazilian population, as well as those of other Latin American nations, were subjected to civil-military dictatorships, health professionals and workers in Brazil engaged with democratization movements and developed initiatives marked by critical reflections and analyses based on emerging theoretical frameworks. These analyses produced a distinct understanding of the health industry, health systems, and the social determinants of health in capitalist societies. In this context, the field of public health emerged, shaped by critical perspectives that differentiated it from the approaches upheld by traditional public health and preventive medicine^2^.

The creation of Abrasco brought together participants from these scientific movements and social struggles, fostering opportunities for dialogue. However, dialogue, although further development is still needed in the disciplinary areas of epidemiology, the humanities, and the social sciences in health, as well as in health planning, administration, and policy^3,4^. Within this context of the struggle for social justice and the consolidation of the Brazilian Unified Health System (*Sistema Único de Saúde* – SUS), Brazilian epidemiology emerged as one of the pillars of public health. Since then, it has evolved in continuous dialogue with the other fields that constitute the discipline — social sciences in health and health policy, planning, and management — while embodying Abrasco's commitments to building a more equitable society and promoting improved living conditions and health for the entire Brazilian population^5^.

Since its inception, Abrasco, through its Epidemiology Commission, has been established to develop master plans for advancing epidemiology in Brazil. The first plan was prepared in 1989^6^ and the fourth in 2005^7^. Over time, these documents have presented fundamental guidelines and proposals that, once implemented, contributed decisively to the consolidation, expansion, and qualification of epidemiology as a strategic disciplinary field within Brazilian public health.

The preparation of the Fifth Strategic Plan for the Development of Epidemiology in Brazil (2025–2029), undertaken 19 years after the IV Plan, occurs in a context marked by events and transformations in Brazil and worldwide that are shaping new scenarios and posing distinct challenges^8^. In recent decades, the expansion of neoliberal policies, within the framework of the financialization of the capitalist mode of production, has resulted in income concentration, increased poverty, job insecurity, widening health inequities, and reduced investment in health and social welfare policies. The COVID-19 pandemic, which unfolded in Brazil under a denialist government, exposed the growing spread and acceptance of anti-vaccine and anti-science views among segments of the population, raising new questions for public health and, in particular, for epidemiology. The present context poses far greater challenges for defending SUS and safeguarding the health of the Brazilian population. Among the central aspects defining the current situation, the climate crisis stands out as an increasingly pressing issue. At the same time, significant technological advances in recent years have opened new perspectives for important applications in the health field, but such innovations must be accompanied by critical evaluations that consider all potentially affected dimensions.

The activities proposed in the four previous master plans contributed to the advancement of epidemiology, underscoring its intrinsic relevance to the field of public health and fostering a successful process of expansion, expressed through its various levels of activity: training, research, and integration with health policies and health services.

In the field of education, notable progress includes the significant expansion of postgraduate programs in collective health/public health, as well as the creation and growth of undergraduate programs in collective health. These programs expanded, albeit unevenly, across different regions of the country, also contributing to the interiorization process.

In the field of research, lines of investigation have multiplied, with results evident in the substantial growth of bibliographic production published in national and international journals and books, as well as in technical-technological outputs, marked by significant advances in methods and techniques.

The quality of the academic publications is comparable to that of those produced globally, with broad coverage of the health problems affecting the Brazilian population. As an example — without diminishing the value of many other contributions — is the coverage of robust epidemiological analyses of the COVID-19 pandemic. It is also important to emphasize the fundamental role of funding agencies — the National Council for Scientific and Technological Development, the Coordination for the Improvement of Higher Education Personnel, and the Research Support Foundations — which, owing to their specific characteristics, have become essential institutions for scientific progress when properly managed.

In the articulation of health policies and services, the participation and effective contributions of the epidemiological community, guided by the master plans, have been significant. The definition of strategic agendas and the monitoring of institutional structures at different levels of the SUS have relied on the active, continuous, and close collaboration of Abrasco, whose work extends beyond the disciplinary field of epidemiology. Likewise, the Association has provided substantial support for the production of knowledge generated within health services.

Despite the significant progress achieved through the implementation of the four previous plans, supported by the Pan American Health Organization (PAHO) and the Ministry of Health, emerging challenges in health necessitate updates across the three levels of action mentioned above. Within a context of unwavering defense of democracy, the National Policy on Science, Technology, and Innovation, and SUS, and in the pursuit of reducing social inequities/inequalities in health, the development of the Fifth Strategic Plan for the Development of Epidemiology in Brazil (2025–2029) represents a fundamental milestone aimed at contributing to the country's progress.

The Fifth Strategic Plan presents a comprehensive, contextualized, and updated assessment of the current health challenges. It takes as its initial reference the synthesis of previous plans, particularly the Fourth Strategic Plan, considering three thematic areas: training; epidemiological research; and the application of epidemiology in health policies, programs, and services. Within each thematic area, the plan identifies new dimensions of previously recognized issues and introduces emerging problems/challenges, aligning with the overarching goal of further consolidating the field of public health.

To uphold the principles of plurality, diversity, inclusion, and democratic leadership in its development, the plan involved the active participation of epidemiologists from all regions of Brazil, representing multiple generations of researchers, faculty, and SUS workers. Additionally, the process sought to ensure gender and racial/ethnic equity, thereby reinforcing the potential for collective and democratic development grounded in epidemiology's commitment to addressing social inequalities in health.

It is anticipated that the Fifth Strategic Plan, substantially supported by the Department of Strategic Actions in Health Surveillance of the Secretariat of Health and Environment Surveillance within the Ministry of Health, will provide a structured framework for impactful actions, stimulate significant theoretical and methodological advances, and, above all, promote effective progress in strengthening SUS and in developing successful, participatory strategies to reduce social inequalities in health.

## FIFTH STRATEGIC PLAN FOR THE DEVELOPMENT OF EPIDEMIOLOGY IN BRAZIL (2025–2029)

After 19 years, the Abrasco Epidemiology Commission has resumed the process of developing a new Master Plan: the Fifth Strategic Plan for the Development of Epidemiology in Brazil for the five-year period 2025–2029.

The development of this plan was structured in several phases. The first phase involved summarizing the discussions on the identification of problems and challenges for epidemiology, conducted at the Workshop I of the Fifth Strategic Plan for the Development of Epidemiology in Brazil, held in Brasília (Federal District – FD), on August 17 and 18, 2023. The second phase focused on systematizing the results of an online survey completed by the community of epidemiologists who participated in the Workshop I, with the aim of identifying strategies to address the problems identified. The third phase, conducted at the Workshop II in Rio de Janeiro (RJ), on September 12 and 13, 2024, consolidated all previous contributions. Detailed information on the activities and profiles of participants involved in developing the Fifth Strategic Plan for the Development of Epidemiology in Brazil (2025–2029) is provided in Appendix 1.

During this period, several remote and in-person meetings were conducted, involving intensive participatory work to achieve the results presented here.

The development of the Fifth Strategic Plan for the Development of Epidemiology in Brazil (2025–2029) was made possible through the dedicated and committed work of a large number of Brazilian epidemiologists. Special recognition is due to the Strategic Plan Working Group of the Abrasco Epidemiology Commission, which, beginning in 2022, planned and coordinated the development process. The active participation of the team from the Secretariat of Health and Environmental Surveillance of the Ministry of Health in this process is also noteworthy. The product presented here represents a synthesis of the many contributions and discussions held over the years and, like previous plans, reflects a vision for the future development of epidemiology in Brazil.

## THEMATIC AREAS: IDENTIFIED PROBLEMS AND PROPOSED ACTIONS

### Thematic Area I: Training in Epidemiology

#### Axis 1: Undergraduate and postgraduate education

Systematized problems:

- Regional inequalities and asymmetries in the distribution and allocation of financial resources for postgraduate programs, both academic and professional, across the country;
- Need for increased exchange and communication between undergraduate and graduate programs in public health, with an emphasis on epidemiology, across the country;
- Courses and training programs exhibit an imbalance between theoretical instruction and the analysis of epidemiological realities, which remain insufficiently explored. This gap has limited students’ capacity to develop a critical understanding of contemporary challenges, thereby hindering the application of theories and concepts to specific contexts;
- Disconnect between epidemiology and other core disciplines that underpin the field of public health has negatively impacted the teaching and learning process;
- Limited integration of epidemiological knowledge into the teaching of topics related to social inequalities in health, as well as environmental education, human rights education, ethnic-racial relations, and issues concerning Afro-Brazilian and Indigenous cultures in undergraduate and graduate programs;
- Limited integration of epidemiological knowledge into clinical practice across various health science disciplines;
- Epidemiology training, at both undergraduate and postgraduate levels, often does not adequately address local and regional needs.
- High fragmentation of postgraduate training, resulting from the structuring of areas of concentration without the establishment of permanent channels for dialogue and knowledge exchange, creating significant barriers between the three disciplinary cores of public health and compromising the comprehensive training essential for epidemiologists in the country;
- Pedagogical strategies often fail to adequately account for the specific skills and competencies of both new and former students, as well as the diverse learning styles and specific objectives of each learning process.

Recommended actions to address the identified problems:

- Promote the strategic and regional expansion of undergraduate programs in public health across the country, in accordance with the National Curricular Guidelines, incorporating the teaching of epidemiology within the undergraduate curriculum in connection with postgraduate programs;
- Promote the establishment of postgraduate programs in public health in underserved regions and areas through targeted funding aimed at reducing inequalities and asymmetries;
- Strengthen solidarity and cooperation between established and emerging postgraduate programs by implementing strategies such as targeted postdoctoral programs for faculty members of programs that are not yet fully established;
- Promote integration between graduate programs to facilitate the sharing of experiences in the training process. This initiative should include the identification of successful practices, analysis of existing limitations, and discussion of remaining challenges, with the aim of enhancing academic and professional development;
- Promote the development of strategies to attract and retain faculty in order to consolidate postgraduate programs and research groups in underserved regions and areas;
- Establish channels that facilitate international cooperation through collaborative networks, emphasizing partnerships among countries of the Global South to promote the exchange of knowledge, resources, and innovation, with a focus on human and social development;
- Explore strategies to restore funding for cooperative initiatives between institutions, integrating these efforts into the academic program evaluation system. Additionally, establish robust mechanisms for the continuous monitoring and evaluation of such initiatives to ensure their sustainability and positive impact on the development of interinstitutional collaborations;
- Ensure that epidemiology training is aligned with other disciplines in public health (humanities and social sciences; health policy, planning, and management), fostering a comprehensive and integrated perspective. This approach seeks to reduce knowledge fragmentation and equip professionals with the skills necessary to address complex public health challenges;
- Expand training programs focused on developing theoretical models and understanding the dynamics of health–disease processes, encompassing everything from research instrument design to the critical analysis of data;
- Promote the inclusion of curricular components grounded in social epidemiology frameworks;
- Promote the development of curricular components in undergraduate and graduate programs that focus on epidemiology training for preparedness, surveillance, and response to events that may constitute public health emergencies of national significance. This initiative aims to equip health professionals to participate effectively in the national response to future disasters and pandemics;
- Promote situational assessments of the epidemiology curricular components in undergraduate and graduate public health programs, evaluating the presence of strategies that integrate these components with other disciplinary cores of public health (human and social sciences; health policy, planning, and management);
- Promote the updating of epidemiology curricula across different training approaches, incorporating a greater practical component, such as through reality-based simulations;
- Promote the establishment of permanent dialogic spaces within Abrasco for discussions on epidemiology training, a key process for expanding and enhancing the exchange between different training strategies to address the country's needs;
- Support the integration of epidemiology into extension curricula as a fundamental initiative, developed in close collaboration between higher education institutions and social or community movements;
- Establish regular spaces for dialogue between faculty and students, at both undergraduate and graduate levels, to enable continuous evaluation of epidemiology training in public health, identify gaps and deficiencies, and propose strategies to address them.

#### Axis 2: Teacher Training

Systematized problems:

- Teacher training in pedagogical strategies and digital technologies for teaching epidemiology remains insufficient, resulting in inadequate preparation for employing innovative teaching methods and integrating digital tools that enhance the learning process. This limitation often leads to reliance on traditional approaches, which may fail to engage students and constrain both the understanding of epidemiological concepts and their practical application;
- Limited training in digital technologies hampers the adaptation to contemporary teaching demands, restricting the preparation of future generations of epidemiologists and thereby compromising their capacity to address current public health challenges;
- Absence of nationally coordinated and integrated initiatives for teacher training in epidemiology.

Recommended actions to address the identified problems:

- Promote research on teaching and didactic methods to advance pedagogical practices, including continuous evaluation of current methods and the discussion of innovative approaches and active strategies that enhance the epidemiology teaching–learning process;
- Encourage increased exchange and sharing of experiences among the various epidemiology training centers across the country;
- Support the development, discussion, and dissemination of active pedagogical methods and strategies for teaching epidemiology, incorporating a wider range of technological resources and aligning with the specific skills and objectives of each learning process;
- Promote the integration of active methodologies as pedagogical strategies in epidemiology curricula, ensuring that educational processes are inclusive, engaging, and transformative;
- Promote training programs for teachers focused on the use of digital information and communication technologies, as part of institutional policies;
- Encourage strategies, such as roundtables and workshops, focused on specific teaching practices for epidemiology in undergraduate programs.

#### Axis 3: Articulation and Integration of Teaching-Service-Research-Community

Systematized problems:

- Insufficient training integrated with health service practice, highlighting a disconnect between theoretical instruction and professional realities;
- Limitations of curricula that fail to consider the specific characteristics of each training area or the need for a transversal approach across different modalities of epidemiology education;
- Limited responsiveness of postgraduate epidemiology training, both academic and professional, to the demands of health services within the SUS care network, particularly concerning surveillance activities;
- Deficient coordination and dialogue between educational institutions and health services, undermining the connection with practical realities and with health professionals in both training and continuing education;
- The absence of theoretical and methodological reflection in the analysis of data from Health Information Systems, as well as in the use of digital information and communication technologies, presents a significant challenge to the effectiveness of public health interventions. This gap has constrained the development of critical, well-founded analyses, limiting health professionals’ ability to generate relevant information for decision-making and strategic planning;
- Limited use of free software in epidemiology education at all levels of training, representing a significant barrier to the modernization and enhanced effectiveness of instruction in this field;
- The careful and critical integration of artificial intelligence (AI) into the healthcare sector is essential. Although AI has the potential to transform patient care, data management, and health research, its implementation must be accompanied by a thorough analysis of ethical, social, and operational implications;
- Limited progress in incorporating digital health technologies in training institutions, contrasting with the increasing demand for their use in health services, surveillance, and reference centers;
- Deficient capacity to translate and disseminate knowledge generated through epidemiology training for specific audiences or for the broader public.

Recommended actions to address the identified problems:

- Promote the inclusion of a health surveillance component in undergraduate general health programs and postgraduate public health courses, with the aim of fostering students’ interest in and knowledge of effective practices for addressing health challenges in the country;
- Encourage practical training opportunities in health services, particularly in health surveillance, within undergraduate and postgraduate public health programs;
- Promote extracurricular internships for undergraduate students that facilitate engagement with epidemiology practice within health services under the SUS and in community settings;
- Promote initiatives that foster closer collaboration and integration between managers and health professionals in training and continuing education programs;
- Incorporate health service needs into the development of bibliographic and technical-technological outputs across different course modalities, ensuring that academic training aligns with the real demands of SUS;
- Promote continuing education strategies in epidemiology targeted at health professionals;
- Promote spaces for theoretical and methodological reflection on the analysis of Health Information Systems and the use of digital information and communication technologies;
- Promote the creation of a publicly accessible portal providing free software dedicated to epidemiology;
- Promote the training of students, managers, and researchers in the skills required to apply methods and techniques for translating and disseminating knowledge generated within society.

### Thematic Area II: Research in Epidemiology

#### Axis 1: Social Inequalities and Health Inequities

Systematized problems:

- Limited research integrating the economic model into theoretical frameworks, methodological approaches, and health impact assessments;
- Limited research and knowledge generation regarding vulnerable populations, including people experiencing homelessness, those deprived of liberty, Indigenous communities, rural/riverside populations, forest and aquatic populations, LGBTQIAPN+ communities, *quilombolas*, and people with disabilities.

Recommended actions to address the identified problems:

- Reinforce the importance of research focusing on the origins and mechanisms of socioeconomic inequalities embedded within the capitalist system, which serve as major drivers of health inequities. Given the current levels of income concentration and the implementation of neoliberal policies, it is essential to expand research to monitor the impact of these processes on poverty, health indicators, and the extent of social inequalities in health;
- In addition to examining the effects of the economic model, research should prioritize the living and health conditions of Brazil's underprivileged, marginalized, and oppressed populations. Particular emphasis should be given to Black and female populations, who have historically faced discrimination, devaluation, and exclusion;
- Promote critical curricular components and regular discussions on the health impacts of economic and social policies, production processes, and structural inequalities, aiming to expand research grounded in robust theoretical models of social determinants of health and integrating perspectives from the various disciplinary cores of public health;
- Promote the development of specific indicators to measure intersectionality and complexity, incorporating transdisciplinary approaches and diverse methodologies, in light of the operational challenges of integrating health inequalities and inequities into analytical models;
- Promote research that fosters critical reflection on the various sociological theories applied to models of social determination of the health-disease process and health conditions;
- Promote the pluralistic, critical, and creative application of research methods in studies on social inequalities in health, engaging the communities under study and using appropriate indicators to measure health outcomes in vulnerable populations with attention to intersectionality. This approach may include partnerships with community organizations, prior consultations, and respect for cultural and traditional knowledge;
- Encourage public policies that support studies — through mechanisms such as public calls, scholarships, and other initiatives — and the dissemination of research results on social inequalities in health in Brazil. This includes attention to regional disparities, emphasizing the impact of macrostructural policies and income concentration on the health and quality of life of vulnerable populations;
- Expand collaboration between research groups and graduate programs on studies of health inequities, promoting the sharing of knowledge, initiatives, tools, and techniques to diversify and enhance research in this area. These initiatives should consider the country's inter- and intraregional inequalities, for example, by organizing scientific events with periodic discussions on the topic;
- Advocate for the development of public policies and academic initiatives, such as targeted funding calls, to support strategic research involving diverse populations in contexts of heightened vulnerability;
- Enhance diversity on editorial boards and propose thematic issues focusing on highly vulnerable population subgroups in scientific journals within the field of public health;
- Promote the provision of courses and the organization of seminars that foster research on highly vulnerable population subgroups;
- Promote and support participatory research methodologies that actively involve the communities under study in their own territories, through partnerships with community organizations and prior consultations, thereby strengthening respect for cultural and traditional knowledge;
- Introduce specialized courses and workshops on research with vulnerable population groups at epidemiology conferences, and promote greater diversity and integration among Abrasco thematic groups related to this topic;
- Reinforce and promote research on highly vulnerable populations using human and social rights as a theoretical framework, with particular emphasis on people with disabilities who experience compounded discrimination, facing both restrictions on fundamental rights and marginalization under the neoliberal economic model, which often undervalues them based on perceived lower productivity.

#### Axis 2: Funding for Research and Research Institutions

Systematized problem:

- Insufficient and unstable scientific funding policies, undermining the democratization, strengthening, expansion, and internationalization of Brazilian research groups.

Recommended actions to address the identified problem:

- Engage with funding agencies and municipal, state, and federal government bodies to advocate for more robust and stable funding policies for epidemiological research, as well as to strengthen funding initiatives through collaboration among countries of the Global South;
- Promote the strengthening of funding policies that support early-career PhDs, preventing attrition from scientific careers and the emigration of talent. This includes the provision of tenure-track or postdoctoral fellowships for collaborative networks, as well as increasing the number of competitive examinations for academic positions at universities, institutes, and foundations, and ensuring adequate infrastructure to support the establishment of research groups;
- Mobilize the scientific community to generate evidence on the impact of public investment in science and technology on national development, knowledge advancement, and the improvement of public policies, in order to support advocacy with funding and government agencies for sustained and robust research resources;
- Encourage funding agencies to account for regional inequalities when issuing research calls, promoting equity and addressing the specific needs of each region and the most vulnerable populations;
- Provide training opportunities for early-career researchers to develop competitive proposals and manage resources effectively, thereby enhancing the capacity to attract national and international funding to support epidemiological research in the country;
- Advocate that the selection of projects and research groups for public funding be conducted primarily through widely publicized calls for proposals, restricting direct hiring of research groups to specific situations, such as public health emergencies;
- Promote discussions on funding for reliable data repositories that comply with the General Personal Data Protection Law and are accessible to researchers and health managers.

#### Axis 3: Theory, Method, and Ethics in Research

Systematized problems:

- Theoretical gaps and methodological limitations in epidemiological research, constraining the development of studies with significant social impact;
- Insufficient multidimensional and interdisciplinary approaches to explore the complexity of health problems and their contexts;
- Use of secondary health data with limited critical reflection and insufficient analysis of its potential and limitations for epidemiological research;
- Restricted access to government databases for research purposes;
- Absence of reliable and secure repositories for storing data generated by epidemiological research, hindering the implementation of open science strategies;
- Insufficient debate on the adequacy of ethical considerations in population-based research.

Recommended actions to address the identified problems:

- Promote the critical training of researchers, emphasizing the importance of studies grounded in robust theoretical reflection and research methods that generate significant knowledge and produce solid evidence to address concrete health problems;
- Promote the creation and strengthening of research networks to disseminate theories and methods, integrating postgraduate programs and research groups from diverse regions and varying levels of development;
- Develop continuing education activities for teachers and researchers to strengthen and innovate epidemiological research methods and theoretical frameworks;
- Promote debate and implement initiatives that reduce the emphasis on scientific productivism, supporting research with both scientific and social relevance, grounded in theoretical and methodological rigor, and encouraging the adoption of good editorial and publishing practices;
- Promote greater integration between research groups and graduate programs across diverse areas and disciplines, and develop specific calls for proposals that require or encourage the formation of research teams with varied expertise, including sociology, anthropology, psychology, public health, economics, and cultural studies;
- Establish working groups to identify strategies for public health and epidemiological research aligned with Brazil's digital health strategy, promoting research, innovation, and the development of AI solutions, with a focus on addressing health problems of relevance to the Brazilian population;
- Promote funding for studies focused on developing epidemiological research tools and evaluating their implementation in health services;
- Promote international cooperation to share new technologies, best practices, and successful public health experiences developed in other national contexts;
- Promote events and courses that train and equip researchers in the effective and innovative use of secondary health data. Encourage undergraduate, graduate, and technical programs to emphasize the importance of analyzing data from Health Information Systems and provide guidance for analyses that foster knowledge advancement and practical application in health services;
- Support the continuous training of surveillance teams and other health professionals in the collection and entry of data into Information Systems, aiming to improve data quality, completeness, and its effective use by health services;
- Promote collaboration among professionals in epidemiology, statistics, humanities and social sciences, data management, and other relevant fields to ensure that secondary data analyses incorporate multiple perspectives and diverse methods and techniques;
- Advocate for the continuous improvement of existing databases in Brazil, particularly by expanding coverage, updating and qualifying information, and standardizing variables to facilitate integration between Ministry of Health Information Systems and other government sectors;
- Engage in dialogue with the Ministry of Health and other government agencies on the need for open, democratic, and transparent access to all epidemiologically relevant secondary data, in compliance with the General Personal Data Protection Law;
- Establish spaces for dialogue among researchers, social oversight bodies, and database operators and managers to ensure a consistent interpretation of the General Personal Data Protection Law, guaranteeing the ethical use of data while enabling appropriate access;
- Promote discussions on the funding and development of reliable and secure national data repositories that comply with the General Personal Data Protection Law and are accessible to researchers and health managers;
- Develop instructional materials and foster open discussions with researchers and managers on the importance of open science, secure data management, and the proper use of repositories;
- Encourage debates on innovative initiatives to enhance ethical standards and procedures, adapting them to various types of observational population studies in epidemiology.

### Thematic Area III: Epidemiology in Health Policies, Programs, and Services

#### Axis 1: National Information Systems and Epidemiological Practices: Analysis of the Health Situation According to Living Conditions

Systematized problems:

- Limited interoperability among the multiple Health Information Systems and technologies used in SUS and other government sectors for epidemiological analysis. Challenges include operational fragmentation, technical and/or semantic restrictions, and the absence of a unique identifier code and standardized citizen identification profile, which reduce access and hinder effective intercommunication and use of available data;
- Insufficient availability and quality of data, as well as limited access to and utilization of Health Information Systems, restricting the ability to analyze the epidemiological dimensions of diseases/injuries/events and their determinants, particularly among the most vulnerable populations across diverse social contexts in the country;
- Restricted access to health surveillance data and healthcare networks, including information from private health plans and services such as clinics, hospitals, laboratories, pharmacies, and vaccination centers;
- Weaknesses in data consolidation within the SUS, epidemiological analyses, and information dissemination, limiting the timely and accurate feedback of critical information for decision-making and public communication;
- Insufficient investment to ensure the necessary infrastructure and qualified personnel for health management, particularly for maintaining, updating, and operating Health Information Systems across different areas of SUS management.

Recommended actions to address the identified problems:

- Within Abrasco, strengthen the establishment of strategic spaces to expand technical-scientific participation in health decision-making bodies of the Brazilian State, supported by the recognition of existing or newly established thematic working groups within the Epidemiology Commission;
- Advocate that the ownership and management of data from National Health Information Systems remain the exclusive responsibility of the public sector within national territory, non-delegable, and guided by ethical, democratic, and sovereign principles;
- Advocate for a national investment plan to consolidate strategic actions ensuring access to health information, as well as its availability and quality, at the federal, state, and municipal levels, through legitimate decision-making spaces and guided by responsible application of the General Personal Data Protection Law in alignment with national priorities;
- Propose that National Information Systems implement a unique identification code for each citizen and standardize the citizen identification profile — minimum data set (including fields for social name, gender identity, sexual orientation, race, ethnicity, and disability status, among others) — while safeguarding the principles of the General Personal Data Protection Law;
- Advocate for timely access to data from the private healthcare system, accounting for the diversity of institutions, services, and health plans, with a primary focus on surveillance activities and also supporting strategic health research, particularly in the context of public health emergencies;
- Recommend actions that support the sustainability of National Information Systems through the National Health Data Network, ensuring technical, operational, and semantic interoperability of coding in an integrated manner and making data accessible to other sectors, such as education, social services, and the environment;
- Contribute to the expansion of technical-scientific knowledge production, ensuring equity in addressing regional inequalities and socially determined conditions, with emphasis on the most vulnerable populations and territories, based on the consolidation and analysis of National Information Systems databases and their intersectoral interfaces;
- Recommend the adoption and enhancement of strategic guidelines and policies for access to health data, grounded in the fundamental principle of the right to information, for data originating from both health surveillance and the Health Care Networks within SUS.

#### Axis 2: Education and Training

Systematized problems:

- Challenges in undergraduate and graduate training in expanding the understanding and application of epidemiology to address infodemics, disinfodemics, denialism, and misinformation, emphasizing a critical appraisal of evidence produced by studies;
- Challenges in promoting more inclusive training processes that consider human diversity, including sexual orientation, gender identity, race, ethnicity, social class, and disability;
- Lack of career pathways and stable employment for health professionals trained in epidemiology who address the needs of SUS public policies and support evidence-based decision-making;
- Insufficient investment in the National Policy for Continuing Education in Health (*Política Nacional de Educação Permanente em Saúde* – PNEPS) to support initiatives that promote epidemiological analysis, impact assessment, use of health situation reports, health communication, and the comprehensive utilization of available databases.

Recommended actions to address the identified problems:

- Strengthen training, continuing education, monitoring, evaluation, supervision, information, and communication strategies within SUS, focusing on enhancing management skills and adopting sound epidemiological practices. These strategies should support the development of relevant indicators and promote consistent analysis of the health situation to inform decision-making, including improving the training of healthcare workers at technical, undergraduate, and graduate levels;
- Expand advocacy efforts and establish technical-scientific committees to implement a public health career within SUS, in accordance with current legal regulations, with a strong epidemiology training foundation across care and surveillance networks. This initiative should strengthen health management while defining competencies, responsibilities, and criteria for each level in management, care, surveillance, prevention, control, research, and teaching activities;
- Support the implementation of PNEPS through initiatives that promote the incorporation of territorial-based epidemiological reasoning and methods in surveillance activities, enhancing the capacity to perform priority epidemiological analyses, including situational assessments, process monitoring and evaluation, and the evaluation of effectiveness, cost-benefit, and impact of health interventions;
- Support the adoption of training practices in technical health courses (secondary level), undergraduate and postgraduate programs (*lato sensu*, including residencies; and *stricto sensu*, including professional master's and doctoral programs), and in continuing education for health workers that address human diversity, including sexual orientation, gender identity, race, ethnicity, social class, and disability, through affirmative action policies and other strategies to overcome persistent social inequalities in the country;
- Propose strengthening innovative training strategies that integrate knowledge of new tools for data systematization and analysis, as well as the translation of technical-scientific knowledge into robust foundations for health information and communication, with a focus on expanding national capacity to respond to infodemics, disinfodemics, denialism, and misinformation.

#### Axis 3: Health Surveillance

Systematized problems:

- Insufficient coordination/integration between healthcare and surveillance within SUS, encompassing all points and levels of the Health Care Networks across life cycles, diseases, injuries, events, and social determinants;
- Limiting the effectiveness of health surveillance components for vulnerable populations, including Indigenous peoples, *quilombolas*, settlers, homeless individuals, migrants, refugees and stateless persons, people deprived of liberty, LGBTQIAPN+ individuals, and itinerant communities such as gypsies and circus performers;
- Failure to incorporate new surveillance strategies, including grassroots and participatory surveillance, genomic surveillance, timely detection of new pathogens/variants, predictive surveillance, and the use of social media for event monitoring;
- Limited incorporation of territorial and local contextual dimensions, which increases challenges in addressing social determinants and risk factors;
- Limiting the availability of simplified information for health management and society, hindering the translation of knowledge (popularization and dissemination) for effective communication and public participation in health.

Recommended actions to address the identified problems:

- Promote the implementation of priority research across different SUS contexts, with strong social participation and a focus on enhancing health surveillance activities in connection with Health Care Networks, valuing transdisciplinary and interepistemic approaches to address social inequalities and inequities;
- Support the development and wide dissemination of strategic indicators for territorially based health surveillance across its various dimensions, including multidimensional indicators integrating social determinant perspectives to define priority areas, with a focus on guiding actions to address diverse challenges and issues identified within SUS;
- Support the adoption of evidence generated by National Information Systems for decision-making within the SUS, grounded on epidemiological methods and techniques, and integrated with territorial-based strategies such as popular, citizen, and participatory surveillance, genomic surveillance, timely detection of new pathogens/variants, predictive surveillance, use of social media for event and rumor monitoring, and the incorporation of new technologies;
- Support the implementation of epidemiological methods and techniques to analyze social inequalities and inequities within SUS, while fostering the transdisciplinary, interepistemic, and intersectoral collaboration necessary for government initiatives addressing these issues from a public health perspective;
- Establish priorities for strengthening, structuring, and organizing the actions of the national network of public health laboratories as an integral component of territorial-based health surveillance;
- Advocate for the expansion of funding for policies and programs involving strategic actions of the health economic and industrial complex to overcome barriers to healthcare access in the country, recognizing health as a human right;
- Advocate for the effective implementation of the National Health Surveillance Policy and the National Workers’ Health Policy, ensuring their integration with the National Primary Care Policy through shared work agendas and intersectoral coordination.

#### Axis 4: Evaluation of Health Programs, Services and Interventions

Systematized problems:

- Weakness of initiatives for monitoring and epidemiologically evaluating the impact of health surveillance models in current national contexts, limiting their use as evidence for effective reformulation;
- Weakness of policies, programs, and integrated actions aimed at structuring and organizing laboratory networks (both public and private) to support the structuring, monitoring, and evaluation of health surveillance activities;
- Insufficient funding for the development of epidemiological monitoring and evaluation activities;
- Limited guidance for professional postgraduate programs (master's and doctoral) to incorporate epidemiological studies on priority national themes, including the implementation, monitoring, and evaluation of health programs, services, and interventions;
- Limited scope of epidemiological perspectives in addressing national demands and priorities in a coordinated manner with the actions of the Ministry of Health.

Recommended actions to address the identified problems:

- Expand and strengthen channels for disseminating epidemiological knowledge in the country, such as journals, technical-scientific events, websites, platforms, and repositories, to publicize successful epidemiology initiatives implemented in different territories and SUS management contexts, prioritizing perspectives grounded in social determinants and dialogue with Brazilian society;
- Promote the development, qualification, valorization, and dissemination of technical and technological outputs related to situational analyses, aiming to propose solutions to challenges in surveillance and care. This includes incorporating training strategies in technical health courses (secondary level), undergraduate and postgraduate courses (*lato sensu*, with emphasis on residencies, and *stricto sensu*, particularly professional master's and doctoral programs), as well as continuing education for workers in SUS territories and services;
- Propose initiatives to strengthen monitoring and evaluation activities, emphasizing a decentralized and integrated system of data and information for surveillance and healthcare networks. These initiatives should prioritize consolidation through participatory processes, respect regional and population diversity, and acknowledge successful experiences, such as those highlighted at the National Exhibition of Successful Experiences in Epidemiology, Prevention, and Disease Control;
- Advocate for the expansion of information technology infrastructure in the SUS care and surveillance network, ensuring adequate equipment, enhanced internet coverage and quality, and the implementation of safe and efficient operational workflows, supported by qualified personnel;
- Advocate for expanded funding for strategic training initiatives to strengthen planning, monitoring, and evaluation capacities based on epidemiological methods, aiming to enhance decision-making at all levels of SUS management. These initiatives should involve educational and research institutions in training professionals through innovative strategies, such as the Education through Work for Health Program, health surveillance programs, and professional-mode postgraduate courses;
- Contribute to the development of methods and techniques for monitoring and participatory evaluation of information production activities and the integration of new technologies across all levels of care, surveillance, and management within SUS.

#### Axis 5: Inclusion of Epidemiology in Intra and Intersectoral Policies

Systematized problems:

- Challenges in implementing epidemiological perspectives and approaches to health inequities, including the inter- and transdisciplinary integration of the three core disciplinary domains of public health;
- Heterogeneity and substantial complexity in the application of epidemiology across different levels of SUS management and between them for evidence-based public policy decision-making;
- Limited recognition and dissemination of successful epidemiological actions implemented within SUS territories and management contexts;
- Limited collaborative initiatives among postgraduate programs (in public health), researchers, institutions, and health services to expand the integration of extension, teaching, service, and community activities as a State policy rather than solely a government initiative.

Recommended actions to address the identified problems:

- Contribute to expanding mobilization and social engagement with broad civil society participation, ensuring representation and diversity through leaders, movements, and social organizations, within Abrasco's sociopolitical decision-making spaces, thereby strengthening successful strategic epidemiological actions implemented across different territories and SUS management contexts;
- Contribute to the creation of spaces that foster greater integration among Abrasco's thematic committees and working groups, aiming to recognize and expand strategic training and communication initiatives within undergraduate and graduate programs focused on solidarity and collaboration. These initiatives should integrate, translate, and disseminate health priorities for Brazilian society through health surveillance actions, including epidemiological practices. They should also promote the integration of extension, teaching, service, and community activities, while valuing democratic, participatory, and legitimate spaces throughout the country;
- Contribute to the expansion of mobilization strategies and the engagement of leaders, Health Council representatives, and social movement actors in education, with the aim of strengthening the National Network for Training in Health Surveillance within SUS, ensuring it remains citizen-oriented, democratic, and participatory.

## Figures and Tables

**Figure 1:**
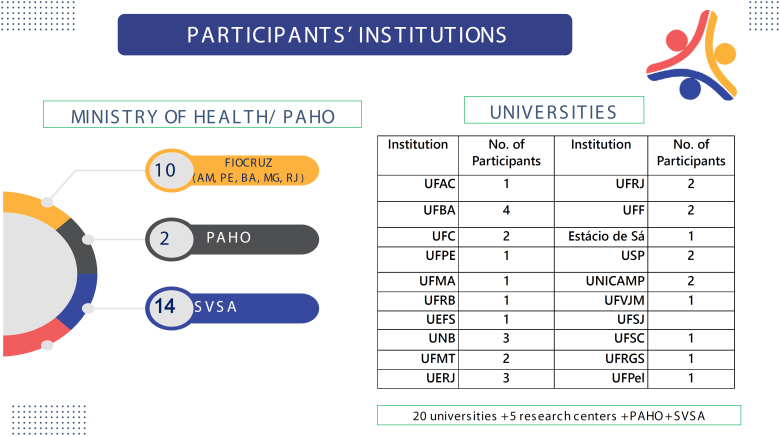
Number of workshop participants by institution of origin.

Fiocruz: Fundação Oswaldo Cruz; PAHOs: Pan Americana Health Organization; SVSA: Secretaria de Vigilância em Saúde e Ambiente; UFAC: Universidade Federal do Acre; UFBA: Universidade Federal da Bahia; UFC: Universidade Federal do Ceará; UFPE: Universidade Federal de Pernambuco; UFMA: Universidade Federal do Maranhão; UFRB: Universidade Federal do Recôncavo Baiano; UEFS: Universidade Estadual de Feira de Santana; UNB: Universidade de Brasília; UFMT: Universidade Federal do Mato Grosso; UERJ: Universidade do Estado do Rio de Janeiro; UFRJ: Universidade Federal do Rio de Janeiro; UFF: Universidade Federal Fluminense; USP: Universidade de São Paulo; Unicamp: Universidade Estadual de Campinas; UFVJM: Universidade Federal dos Vales do Jequitinhonha e Mucuri; UFSJ: Universidade Federal de São João del-Rei; UFSC: Universidade Federal de Santa Catarina; UFRGS: Universidade Federal do Rio Grande do Sul; UFPEl: Universidade Federal de Pelotas.

## Data Availability

The complete dataset supporting the findings of this study is available upon request from the corresponding author, Tânia Maria de Araújo. The dataset is not publicly available due to the presence of sensitive information with potential to identify participants. Data collection was conducted in collaborative contexts and requires ethical and responsible handling. Access is therefore restricted to ensure the privacy of participants and to comply with principles of personal data protection. Consequently, access is limited due to ethical requirements related to participant confidentiality.

## APPENDIX 1.

### Additional information on the development process of the Fifth Strategic Plan for the Development of Epidemiology in Brazil (2025–2029) and the profile of participants involved.

The development process of the Fifth Strategic Plan for the Development of Epidemiology in Brazil (2025–2029) was structured in two phases:

- Phase 1: Diagnosis of the main issues assessed in three thematic areas: I. Education; II. Research; III. Epidemiology in Health Policies, Programs, and Services;
- Phase 2: Proposal of actions and strategies to address the priority issues identified in phase 1.

In each phase, an electronic survey was conducted, preliminary technical reports were prepared, and an in-person workshop was held for discussion and decision-making. The first survey aimed to identify priority problems in each axis, serving as the basis for the reports that guided the first workshop.

In Brasília, on August 17–18, 2023, the **Workshop I** for the development of the **Fifth Strategic Plan for the Development of Epidemiology in Brazil (2025–2029)** was held, thus completing **phase 1** of the development process. The diagnosis of the problems was consolidated in a technical report, derived from the discussions and reporting of the groups in the three thematic areas mentioned above. Figure 1 presents the general characteristics of the group of workshop participants

Based on the information from the technical report of **phase 1** (definition of priority problems), a survey was designed to collect suggestions for actions to overcome the problems identified. Thus, an electronic form was sent to the participants of the Workshop I to record suggestions for strategies to address these issues.

As preparation for the Workshop II, a preliminary technical report was prepared with suggestions collected through the electronic survey. This report brought together contributions intended to guide discussions and define actions for the development of epidemiology over the following five years, which were discussed and approved during Workshop II.

Workshop II was held on September 12 and 13, 2024, in Rio de Janeiro, with the purpose of implementing **Phase 2**, focused on the discussion and definition of proposed actions.

The process of developing the Master Plan involved a broad group of epidemiologists from all regions of the country. Table 1 presents the profile of participants engaged in at least one of the stages carried out, including electronic surveys and workshops.

**Table 1:** Profile of participants in the different stages of the development process of the Fifth Strategic Plan for the Development of Epidemiology in Brazil (2025–2029). Brazil, 2023–2024.

Characteristics		%
Region		
	North	4.5
	Northeast	20.9
	Central-West	18.2
	Southeast	40.9
	South	15.5
**General characteristics of informants**		
Gender		
	Cisgender woman	60.7
	Cisgender man	36.4
	*Travesti*	1.0
	Prefers not to inform	1.9
Age range (years)*		
	Up to 41	26.7
	42–50	22.9
	51–64	25.7
	65 or more	24.8
Race/color		
	White	77.5
	Black and brown	21.5
	Yellow (Asian)	1.0
Undergraduate degree**		
	Medicine	28.7
	Nutrition	13.9
	Dentistry	7.4
	Nursing	6.5
	Physiotherapy	6.5
	Pharmacy	5.6
	Physical Education	4.6
	Statistics	4.6
Main workplace		
	Public university	66.6
	Public health management	21.2
	Research center	9.1
	Private university	3.1
Time working as an epidemiologist (years)		
	Up to 10	25.7
	11–15	19.0
	16–25	26.7
	26 or more	28.6
Current field of work***		
	Academic/teaching activity	78.8
	Research activity	84.8
	Health services activity	36.4

* Mean age: 51.2 years; median: 51.0 years; minimum age: 29 years; maximum age: 79 years;

** other reported undergraduate degrees: Public Health (n=3; 2.8%), Biomedicine (n=3; 2.8%), Biological Sciences (n=3; 2.8%), Speech Therapy (n=3; 2.8%); Social Sciences (n=1; 1.9%), Engineering (n=1; 1.9%), Geography (n=1; 1.9%), Mathematics (n=1; 1.9%), Psychology (n=1; 1.9%), Social Work (n=1; 1.9%);

*** variable with multiple responses.
