# Supplementary material for: Fifth Strategic Plan for the Development of Epidemiology in Brazil (2025–2029)
Source: Rev Bras Epidemiol. 2025 Nov 17;28(Suppl 1):e250001supl1. doi: 10.1590/1980-549720250001.supl.1 (PMC12622983; doi:10.1590/1980-549720250001.supl.1)
Supplement: Translation

# Translation: V Plano Diretor para o Desenvolvimento da Epidemiologia no Brasil (2025–2029)

**DOI:** 10.1590/1980-549720250001.supl.1.2

**Authors:** Tânia Maria de Araújo, Enirtes Caetano Prates Melo, Claudia Leite de Moraes, Laio Magno, Ana Paula Muraro, Maria de Jesus Mendes da Fonseca, Alberto Novaes Ramos, Luana Giatti Gonçalves, Margareth Guimarães Lima, Ligia Regina de Oliveira, Moisés Goldbaum, Joilda Silva Nery, Maria Rita Donalisio, Maria da Glória Lima Cruz Teixeira, Katia Vergetti Bloch, Antonio Fernando Boing, Guilherme Loureiro Werneck, *Comissão de Elaboração do Plano Diretor, Área Temática I: Formação em Epidemiologia, Amanda de Moura Souza, Edna Massae Yokoo, Elisabeth Carmen Duarte, Emanuele Souza Marques, Janaína dos Santos Motta, João Roberto Cavalcante Sampaio, Juan José Cortez Escalante, Katia Crestine Poças, Lívia Teixeira Souza Maia, Michael Eduardo Reichenheim, Naomar Monteiro de Almeida-Filho, Paola Barbosa Marchesini, Rodrigo Citton Padilha dos Reis, Sérgio Viana Peixoto, Área Temática II: A Pesquisa em Epidemiologia, Ana Paula Nogueira Nunes, Andréia Moreira de Andrade, Antônio Augusto Moura da Silva, Carolina Geralda Alves, Felipe Guimarães Tavares, Fernando José Herkrath, Isis Polianna Silva Ferreira de Carvalho, Jesem Douglas Yamall Orellana, Ligia Regina Franco Sansigolo Kerr, Maria Amélia de Sousa Mascena Veras, Maria Cynthia Braga, Maria do Carmo Leal, Marilisa Berti de Azevedo Barros, Mauricio Lima Barreto, Paloma de Sousa Pinho, Paulo Rossi Menezes, Sandhi Maria Barreto, Vinícius Silva Belo, Wildo Navegantes de Araújo, Área Temática Epidemiologia em Políticas, Programas e Serviços de Saúde, Alícia Krüger, Amarílis Bahia Bezerra, Bárbara Campos Silva Valente, Carmen Silvia Bruniera Domingues, Claudio Maierovitch Pessanha Henriques, Ethel Leonor Noia Maciel, Fatima Sonally Sousa Gondim, Gerusa Belo Gibson dos Santos, Gulnar Azevedo e Silva, Gustavo Laine Araújo de Oliveira, Heleno Rodrigues Corrêa, Luciana de Carvalho Penna, Maria Juliana Moura Corrêa, Marli Souza Rocha, Maryane Oliveira Campos, Olavo de Moura Fontoura, Sheila Maria Alvim de Matos, Walter Massa Ramalho

**Affiliations:** I Universidade Estadual de Feira de Santana, Núcleo de Epidemiologia – Feira de Santana (BA), Brasil.; II Fundação Oswaldo Cruz, Escola Nacional de Saúde Pública – Rio de Janeiro (RJ), Brasil.; III Universidade do Estado do Rio de Janeiro, Instituto de Medicina Social – Rio de Janeiro (RJ), Brasil.; IV Universidade do Estado da Bahia, Departamento de Ciências da Vida – Salvador (BA), Brasil.; V Universidade Federal de Mato Grosso, Instituto de Saúde Coletiva – Cuiabá (MT), Brasil.; VI Universidade Federal do Ceará, Faculdade de Medicina – Fortaleza (CE), Brasil.; VII Universidade Federal de Minas Gerais, Faculdade de Medicina – Belo Horizonte (MG), Brasil.; VIII Universidade Estadual de Campinas, Faculdade de Ciências Médicas – Campinas (SP), Brasil.; IX Universidade de São Paulo, Faculdade de Medicina – São Paulo (SP), Brasil.; X Universidade Federal da Bahia, Instituto de Saúde Coletiva – Salvador (BA), Brasil.; XI Universidade Federal do Rio de Janeiro, Instituto de Estudos em Saúde Coletiva – Rio de Janeiro (RJ), Brasil.; XII Universidade Federal de Santa Catarina, Departamento de Saúde Pública – Florianópolis (SC), Brasil.; XIII Ministério da Saúde, Secretaria de Vigilância em Saúde e Ambiente – Brasília (DF), Brasil.; Universidade Federal do Rio de Janeiro - Rio de Janeiro (RJ), Brasil.; Universidade Federal Fluminense - Niterói (RJ), Brasil.; Universidade de Brasília - Brasília (DF), Brasil.; Universidade do Estado do Rio de Janeiro - Rio de Janeiro (RJ), Brasil.; Universidade Federal de Pelotas - Pelotas (RS), Brasil.; Ministério da Saúde, Secretaria de Vigilância em Saúde e Ambiente - Brasília (DF), Brasil.; Organizacão Pan-Americana da Saúde - Brasília (DF), Brasil.; Organização Mundial da Saúde - Brasília (DF), Brasil.; Universidade de Brasília - Brasília (DF), Brasil.; Universidade Federal de Pernambuco - Vitória de Santo Antão (PE), Brasil.; Universidade do Estado do Rio de Janeiro - Rio de Janeiro (RJ), Brasil.; Universidade Federal da Bahia - Salvador (BA), Brasil.; Ministério da Saúde, Secretaria de Vigilância em Saúde e Ambiente - Brasília (DF), Brasil.; Universidade Federal do Rio Grande do Sul - Porto Alegre (RS), Brasil.; Fundação Oswaldo Cruz - Belo Horizonte (MG), Brasil.; Universidade Federal dos Vales do Jequitinhonha e Mucuri - Diamantina (MG), Brasil.; Universidade Federal do Acre - Rio Branco (AC), Brasil.; Universidade Federal do Maranhão - São Luís (MA), Brasil.; Ministério da Saúde, Secretaria de Vigilância em Saúde e Ambiente - Brasília (DF), Brasil.; Universidade Federal Fluminense - Niterói (RJ), Brasil.; Fundação Oswaldo Cruz - Manaus (AM), Brasil.; Ministério da Saúde, Secretaria de Vigilância em Saúde e Ambiente - Brasília (DF), Brasil.; Fundação Oswaldo Cruz - Manaus (AM), Brasil.; Universidade Federal do Ceará - Fortaleza (CE), Brasil.; Faculdade de Ciências Médicas da Santa Casa de São Paulo - São Paulo (SP), Brasil.; Fundação Oswaldo Cruz - Recife (PE), Brasil.; Fundação Oswaldo Cruz - Rio de Janeiro (RJ), Brasil.; Universidade Estadual de Campinas - Campinas (SP), Brasil.; Universidade Federal da Bahia - Salvador (BA), Brasil.; Universidade Federal do Recôncavo da Bahia - Santo Antônio de Jesus (BA), Brasil.; Universidade de São Paulo - São Paulo (SP), Brasil.; Universidade Federal de Minas Gerais - Belo Horizonte (MG), Brasil.; Universidade Federal de São João Del-Rei - Divinópolis (MG), Brasil.; Universidade de Brasília - Brasília (DF), Brasil.; Ministério da Saúde, Secretaria de Vigilância em Saúde e Ambiente - Brasília (DF), Brasil.; Organização Pan-Americana da Saúde - Brasília (DF), Brasil.; Fundação Oswaldo Cruz - Rio de Janeiro (RJ), Brasil.; Secretaria de Estado da Saúde de São Paulo - São Paulo (SP), Brasil.; Fundação Oswaldo Cruz - Brasília (DF), Brasil.; Ministério da Saúde, Secretaria de Vigilância em Saúde e Ambiente - Brasília (DF), Brasil.; Universidade Federal do Espírito Santo - Vitória (ES), Brasil.; Ministério da Saúde, Secretaria de Vigilância em Saúde e Ambiente - Brasília (DF), Brasil.; Universidade Federal do Rio de Janeiro - Rio de Janeiro (RJ), Brasil.; Universidade Estadual do Rio de Janeiro - Rio de Janeiro (RJ), Brasil.; Ministério da Saúde, Secretaria de Vigilância em Saúde e Ambiente - Brasília (DF), Brasil.; Universidade de Brasília - Brasília (DF), Brasil.; Ministério da Saúde, Secretaria de Vigilância em Saúde e Ambiente - Brasília (DF), Brasil.; Ministério da Saúde, Secretaria de Vigilância em Saúde e Ambiente - Brasília (DF), Brasil.; Ministério da Saúde, Secretaria de Vigilância em Saúde e Ambiente - Brasília (DF), Brasil.; Ministério da Saúde, Secretaria de Vigilância em Saúde e Ambiente - Brasília (DF), Brasil.; Ministério da Saúde, Secretaria de Vigilância em Saúde e Ambiente - Brasília (DF), Brasil.; Universidade Federal da Bahia - Salvador (BA), Brasil.; Universidade de Brasília - Brasília (DF), Brasil.

**Keywords:** Epidemiologia, Planejamento estratégico, Educação em saúde, Pesquisa, Políticas de saúde

## Abstract

A Associação Brasileira de Saúde Coletiva (Abrasco) tem sido protagonista na consolidação da saúde coletiva no Brasil desde sua fundação. Após 19 anos, a Comissão de Epidemiologia da Abrasco retomou seu planejamento com a elaboração do V Plano Diretor para o Desenvolvimento da Epidemiologia no Brasil (2025–2029). Esse plano surgiu em um contexto desafiador, marcado por desigualdades sociais, negacionismo científico, pandemia de COVID-19, crise climática e desinvestimento em políticas públicas, reforçando a necessidade de atualização da epidemiologia brasileira. A elaboração do plano ocorreu em múltiplas etapas, incluindo inquéritos *online*, debates e oficinas presenciais. A Oficina I (Brasília, 2023) identificou desafios prioritários, e a Oficina II (Rio de Janeiro, 2024) definiu estratégias de enfrentamento. O processo de construção foi pautado pela participação qualificada de epidemiologistas de todas as regiões do país e fundamentado em princípios de pluralidade, diversidade, equidade de gênero e raça/etnia. O plano destaca a epidemiologia em três áreas temáticas: formação; pesquisa; e políticas, programas e serviços de saúde. O Grupo de Trabalho Plano Diretor da Comissão de Epidemiologia, com apoio da Secretaria de Vigilância em Saúde e Ambiente, do Ministério da Saúde, conduziu o processo. Esse suplemento da *Revista Brasileira de Epidemiologia* apresenta o V Plano Diretor para o Desenvolvimento da Epidemiologia no Brasil (2025–2029), contribuições para debates de alguns temas centrais e aprofundamento de dimensões estratégicas e operacionais. O V Plano Diretor para o Desenvolvimento da Epidemiologia no Brasil (2025–2029) expressa uma visão coletiva de futuro com vistas ao fortalecimento da epidemiologia enquanto disciplina central no campo da saúde coletiva e reafirma seu compromisso com a equidade, a justiça social e a consolidação do Sistema Único de Saúde no Brasil.

## APRESENTAÇÃO

Há 45 anos, nascia a Associação Brasileira de Saúde Coletiva (Abrasco) — à época, Associação Brasileira de Pós-Graduação em Saúde Coletiva. Criada por expressivos militantes das áreas de saúde pública e de medicina social e preventiva, a Abrasco consolidou-se como um lócus privilegiado na luta pela reforma sanitária, articulando-se com os movimentos sociais que reivindicavam a democratização do país^1^. No período em que a população brasileira e as de outros países latino-americanos estavam submetidas a ditaduras civil-militares, profissionais, trabalhadores e trabalhadoras do campo da saúde no Brasil integravam os movimentos sociais pela democratização e desenvolviam trabalhos com reflexões críticas e análises fundamentadas em novos referenciais teóricos. Essas análises geraram um entendimento diferenciado sobre a indústria da saúde, os sistemas de saúde e os determinantes da saúde das populações nas sociedades capitalistas. Nascia, assim, o campo da saúde coletiva, construído com base em visões críticas e diferenciando-se das concepções que se sustentavam na saúde pública tradicional e na medicina preventiva^2^.

A criação da Abrasco propiciou a agregação dos participantes desses movimentos científicos e da luta social e abriu nichos para a interlocução, ainda que merecedora de aprimoramento, hoje em dia, dos campos disciplinares da epidemiologia, ciências humanas e ciências sociais em saúde, planejamento, administração e políticas em saúde^3,4^. Foi nesse contexto de luta por justiça social e fortalecimento do Sistema Único de Saúde (SUS) que a epidemiologia brasileira se consolidou como um dos pilares da saúde coletiva. Desde então, desenvolveu-se em permanente diálogo com os demais campos que integram a área — as ciências sociais em saúde e as políticas, planejamento e gestão em saúde —, incorporando os compromissos assumidos pela Abrasco: a construção de uma sociedade mais equitativa e a promoção de melhores condições de vida e saúde para toda a população brasileira^5^.

A Abrasco, por meio de sua Comissão de Epidemiologia, desde seus primórdios, tem se organizado com o objetivo de elaborar planos diretores para o Desenvolvimento da Epidemiologia no Brasil. O primeiro plano foi elaborado em 1989^6^ e o quarto em 2005^7^. Esses documentos, ao longo do tempo, apresentaram diretrizes e proposições fundamentais que, uma vez implementadas, contribuíram de forma decisiva para a consolidação, expansão e qualificação da epidemiologia como campo disciplinar estratégico no escopo da saúde coletiva brasileira.

A elaboração deste V Plano Diretor para o Desenvolvimento da Epidemiologia no Brasil (2025–2029), passados 19 anos da elaboração do IV plano, se dá em um contexto no qual acontecimentos e mudanças de impacto no Brasil e no mundo desenham novos cenários e trazem diferentes desafios^8^. Nas últimas décadas, a expansão das políticas neoliberais, no contexto de financeirização do modo de produção capitalista, implicou concentração de renda, aumento da pobreza, precarização do trabalho, aprofundamento das iniquidades sociais em saúde e desinvestimento em políticas de saúde e bem-estar social. A pandemia de COVID-19, por sua vez, que ocorreu no Brasil durante um governo negacionista, revelou o avanço da difusão e da aderência de segmentos da população a concepções antivacina e anticiência que colocam novas questões para o campo da saúde coletiva e, especialmente, para a epidemiologia. O contexto atual impõe desafios muito maiores à defesa do SUS e à proteção da saúde da população brasileira. Entre os aspectos centrais que definem a atualidade, destaca-se, como um problema crescente, a crise climática. Os grandes avanços tecnológicos dos últimos anos abrem novas perspectivas para importantes aplicações no campo da saúde, mas essas inovações devem ser acompanhadas de avaliações críticas que considerem todas as dimensões que podem ser impactadas.

As atividades propostas pelos quatro planos diretores anteriores contribuíram para que a epidemiologia, ressaltando sua pertinência intrínseca ao campo da saúde coletiva, experimentasse um bem-sucedido processo de expansão expresso nos seus respectivos níveis de atuação: formação, pesquisa e articulação às políticas em saúde e aos serviços de saúde.

Na formação, pode-se salientar a notável expansão dos programas de pós-graduação em saúde coletiva/saúde pública e a criação e a expansão dos cursos de graduação em saúde coletiva. Os programas expandiram-se, embora de forma desigual, pelas diversas regiões do país, alcançando também processos de interiorização.

Na pesquisa, as linhas de investigação multiplicaram-se, e os resultados são evidentes tanto pelo significativo crescimento da produção bibliográfica registrada em periódicos e livros nacionais e internacionais quanto pela produção técnico-tecnológica, com importantes avanços em métodos e técnicas.

A qualidade dos trabalhos publicados equipara-se à qualidade daqueles produzidos globalmente, com ampla cobertura dos problemas de saúde da população brasileira. A título de exemplo, sem desmerecer outros tantos, citamos a cobertura de análises epidemiológicas contundentes da pandemia de COVID-19. Vale destacar o papel fundamental das agências de fomento — Conselho Nacional de Desenvolvimento Científico e Tecnológico, Coordenação de Aperfeiçoamento de Pessoal de Nível Superior e Fundações de Apoio à Pesquisa —, que, pelas suas especificidades, vêm se constituindo, na medida de sua correta condução, em imprescindíveis instâncias para o progresso científico.

Na articulação às políticas de saúde e aos serviços de saúde, a participação e a contribuição efetiva da comunidade epidemiológica, orientadas pelas diretrizes dos planos diretores, têm sido expressivas. Nesse sentido, a definição de pautas estratégicas e o acompanhamento das estruturas institucionais nos diferentes níveis do SUS contam com a colaboração ativa, contínua e próxima da Abrasco, e salienta-se que essa atuação não se restringe ao campo disciplinar da epidemiologia. Da mesma forma, o apoio à produção de conhecimentos gerados no âmbito dos serviços tem encontrado, por parte dessa associação, substanciais colaborações na sua condução.

Não obstante os significativos avanços alcançados com a implementação dos quatros planos anteriores, apoiados pela Organização Pan-Americana da Saúde (Opas) e pelo Ministério da Saúde, os novos desafios e questões que a saúde apresenta exigem a necessária atualização nos três níveis de atuação reportados anteriormente. Em um contexto de defesa irrestrita da democracia, da Política Nacional de Ciência, Tecnologia e Inovação e do SUS, na busca de superação de iniquidades/desigualdades sociais em saúde, a construção/elaboração do V Plano Diretor para o Desenvolvimento da Epidemiologia no Brasil (2025–2029) constitui um marco fundamental com foco na contribuição para os avanços no país.

Este V Plano Diretor apresenta diagnóstico amplo, contextualizado e atualizado dos problemas de saúde enfrentados. Recupera, como referencial inicial, a síntese dos planos anteriores, especialmente do IV Plano Diretor, considerando as três áreas temáticas: formação; pesquisa em epidemiologia; e epidemiologia em políticas, programas e serviços de saúde. Reconhece, em cada uma dessas áreas temáticas, novas dimensões dos problemas previamente identificados e insere novos problemas/desafios, alinhando-se no objetivo maior, de consolidar cada vez mais o campo da saúde coletiva.

Com o intuito de atender aos princípios de pluralidade, diversidade, inclusão e condução democrática na elaboração do plano, o processo contou com a participação ativa de epidemiologistas de todas as regiões brasileiras, representando diferentes gerações de pesquisadores(as), docentes e trabalhadores(as) do SUS. Além disso, buscou garantir equidade de gênero e de raça/etnia, fortalecendo as perspectivas de construção coletiva e democrática sustentadas no compromisso da epidemiologia com o combate às desigualdades sociais em saúde.

Esperamos que este V Plano Diretor, apoiado substancialmente pelo Departamento de Ações Estratégicas em Vigilância em Saúde da Secretaria de Vigilância em Saúde e Ambiente, do Ministério da Saúde, estruture um conjunto profícuo de ações, estimule o desenvolvimento significativo teórico e metodológico e, sobretudo, impulsione avanços efetivos no fortalecimento do nosso SUS e na construção de estratégias exitosas e participativas na redução das desigualdades sociais em saúde.

## V PLANO DIRETOR PARA O DESENVOLVIMENTO DA EPIDEMIOLOGIA NO BRASIL (2025–2029)

Após 19 anos, a Comissão de Epidemiologia da Abrasco retoma o processo de construção de mais um Plano Diretor: o V Plano Diretor para o Desenvolvimento da Epidemiologia no Brasil, quinquênio 2025–2029.

O processo de elaboração deste plano foi estruturado em diferentes momentos. O primeiro deles consistiu na sumarização dos debates sobre o levantamento de problemas e desafios para a epidemiologia realizados na I Oficina do V Plano Diretor para o Desenvolvimento da Epidemiologia no Brasil, em Brasília (DF), nos dias 17 e 18 de agosto de 2023. No segundo momento, foi feita a sistematização da consulta por inquérito *on-line* à comunidade de epidemiologistas que participou da I Oficina. O inquérito objetivou elencar estratégias de enfrentamento dos problemas identificados. O terceiro momento, ocorrido na II Oficina, que aconteceu no Rio de Janeiro (RJ), nos dias 12 e 13 de setembro de 2024, sistematizou todas as contribuições anteriores. Informações das atividades e perfil dos(as) participantes na elaboração do V Plano Diretor para o Desenvolvimento da Epidemiologia no Brasil (2025–2029) estão no Anexo 1.

Ao longo de todo o período, foram realizadas diversas reuniões remotas e presenciais, com um intenso trabalho participativo para o alcance dos resultados aqui apresentados.

A elaboração do V Plano Diretor para o Desenvolvimento da Epidemiologia no Brasil (2025–2029) somente foi possível pelo trabalho dedicado e compromissado de um grande número de epidemiologistas brasileiros(as). Cabe registrar os esforços do Grupo de Trabalho Plano Diretor da Comissão de Epidemiologia da Abrasco, que, a partir de 2022, planejou e conduziu o processo de elaboração deste plano. Ressalta-se, ainda, a participação ativa da equipe da Secretaria de Vigilância em Saúde e Ambiente, do Ministério da Saúde, nesse processo. O produto aqui registrado é uma síntese das muitas contribuições e dos debates realizados no decorrer desses anos e, assim como os demais planos, expressa uma visão de futuro para o desenvolvimento da epidemiologia brasileira.

## ÁREAS TEMÁTICAS: PROBLEMAS IDENTIFICADOS E AÇÕES PROPOSTAS

### Área temática I: Formação em Epidemiologia

#### Eixo 1: Ensino de graduação e pós-graduação

Problemas sistematizados:

- Desigualdades e assimetrias regionais na distribuição e na alocação de recursos financeiros nos programas de pós-graduação tanto na modalidade acadêmica quanto na profissional no país;
- Necessidade de mais intercâmbio e comunicação entre os programas de graduação e de pós-graduação no campo da saúde coletiva, com ênfase na epidemiologia, no país;
- Cursos e programas de formação apresentam desequilíbrio entre a teoria e a análise da realidade epidemiológica, que permanece insuficientemente explorada. Essa lacuna tem limitado a capacidade de estudantes no desenvolvimento de compreensão crítica de desafios contemporâneos, dificultando sua habilidade de aplicar teorias e conceitos em contextos específicos;
- Dissociação entre a epidemiologia e outros núcleos disciplinares que compõem as bases do campo da saúde coletiva, impactando negativamente no processo de ensino-aprendizagem;
- Incorporação incipiente do conhecimento epidemiológico no tratamento de conteúdos relacionados às desigualdades sociais em saúde, assim como em temas de educação ambiental, educação em direitos humanos, relações étnico-raciais e questões relativas às culturas afro-brasileira e indígena nos cursos de graduação e pós-graduação;
- Incipiente incorporação do conhecimento epidemiológico nas práticas clínicas nas diferentes áreas das ciências da saúde;
- A formação em epidemiologia, tanto na graduação quanto na pós-graduação, com frequência não consegue atender adequadamente às necessidades locais e regionais.
- Intensa fragmentação da formação na pós-graduação, decorrente da estruturação das áreas de concentração, sem a criação de canais permanentes de diálogo e troca de saberes, impondo barreiras significativas à interlocução entre os três núcleos disciplinares da saúde coletiva, comprometendo a formação abrangente desejada e tão necessária para epidemiologistas no país;
- As estratégias pedagógicas não consideram adequadamente habilidades nem competências específicas do perfil de ingressantes e egressos(as), tampouco as diferentes maneiras de aprender e os objetivos específicos de cada processo de aprendizagem.

Ações recomendadas para o enfrentamento dos problemas identificados:

- Estimular a abertura estratégica e regionalizada de bacharelados em saúde coletiva no país, seguindo os referenciais das Diretrizes Curriculares Nacionais, que incluam o ensino da epidemiologia no âmbito da graduação em interface com programas de pós-graduação;
- Estimular a abertura de programas de pós-graduação em saúde coletiva em regiões e áreas deficitárias por meio de financiamento específico com foco na redução de desigualdades e assimetrias;
- Fortalecer ações de solidariedade e cooperação entre programas de pós-graduação consolidados e não consolidados, por intermédio da promoção de diferentes estratégicas, como pós-doutorados específicos para docentes de programas ainda não consolidados;
- Fomentar a integração entre programas de pós-graduação com o intuito de promover o compartilhamento de experiências nos processos formativos. Esse esforço deve incluir a identificação de práticas bem-sucedidas, a análise das limitações enfrentadas e a discussão dos desafios ainda pendentes, com o objetivo de aprimorar as formações acadêmica e profissional;
- Incentivar o desenvolvimento de estratégias de atração e fixação de docentes para a consolidação dos programas de pós-graduação e grupos de pesquisa em regiões e áreas deficitárias;
- Criar canais que favoreçam a cooperação internacional por meio de redes colaborativas, destacando-se a relevância das parcerias entre países do Sul Global para promover trocas de conhecimento, recursos e inovação, com ênfase no desenvolvimento humano e social;
- Estudar formas de explorar estratégias para a retomada do financiamento de iniciativas de cooperação entre instituições, integrando-as ao sistema de avaliação dos programas acadêmicos. Além disso, é fundamental estabelecer mecanismos sólidos de monitoramento e avaliação contínua dessas iniciativas, assegurando sua sustentabilidade e seu impacto positivo no desenvolvimento de colaborações interinstitucionais;
- Garantir formação em epidemiologia que esteja alinhada às demais formações discursivas no campo da saúde coletiva (ciências humanas e sociais; política, planejamento e gestão em saúde), promovendo uma visão ampla e integrada. Essa perspectiva formativa visa reduzir a fragmentação do conhecimento, permitindo que profissionais desenvolvam capacidade robusta para enfrentar problemas complexos em saúde pública;
- Ampliar a oferta de programas de formação com foco na construção de modelos teóricos e compreensão das dinâmicas envolvidas nos processos saúde-doença que orientem desde o planejamento dos instrumentos de pesquisa até a análise crítica dos dados;
- Estimular a oferta de componentes curriculares com abordagem fundamentada nos referenciais da epidemiologia social;
- Estimular a criação de componentes curriculares de ensino da epidemiologia em cursos de graduação e pós-graduação voltados para preparação, vigilância e resposta a eventos que podem vir a constituir emergências em saúde pública, de relevância nacional. O foco direciona-se à formação de profissionais de saúde que atuarão na resposta nacional a futuros desastres e pandemias;
- Incentivar a realização de diagnósticos situacionais acerca dos componentes curriculares de epidemiologia nos cursos de graduação e pós-graduação em saúde coletiva, avaliando a existência de estratégias para a promoção de integração desse componente com outros que trazem os demais núcleos disciplinares constitutivos do campo da saúde coletiva (ciências humanas e sociais; política, planejamento e gestão em saúde);
- Incentivar a atualização da matriz curricular dos cursos de formação nas diferentes perspectivas formativas em epidemiologia, com a inserção de maior carga horária prática, como por meio de simulações baseadas na realidade;
- Estimular a criação de espaços dialógicos permanentes na Abrasco para debates sobre a formação em epidemiologia, processo fundamental para ampliar e intensificar o intercâmbio entre diferentes estratégias formativas para alcance das necessidades do país;
- Apoiar a inserção da epidemiologia na curricularização da extensão como iniciativa fundamental em um processo desenvolvido com estreita colaboração entre instituições de ensino superior e movimentos sociais ou comunitários;
- Criar espaços regulares de diálogo entre docentes e discentes, tanto na graduação quanto na pós-graduação, para avaliação contínua da formação em epidemiologia no campo da saúde coletiva, identificação de lacunas e insuficiências e proposição de soluções para superá-las.

#### Eixo 2: Formação Docente

Problemas sistematizados:

- A formação docente em estratégias pedagógicas e tecnologias digitais para o ensino da epidemiologia é insuficiente, resultando em preparo inadequado para utilização de métodos de ensino inovadores e para integração de ferramentas digitais que enriqueçam o processo de aprendizagem. Essa limitação leva à dependência de abordagens tradicionais que, muitas vezes, não engajam discentes e limitam a compreensão de conceitos epidemiológicos, bem como a sua aplicação prática;
- A limitada formação em tecnologias digitais dificulta a adaptação a demandas contemporâneas do ensino, restringindo a formação de gerações futuras de epidemiologistas, o que compromete as possibilidades de enfrentamento dos desafios atuais em saúde pública;
- Ausência de iniciativas de base nacional e integradas com foco na formação docente em epidemiologia.

Ações recomendadas para o enfrentamento dos problemas identificados:

- Promover estudos sobre a docência e seus aspectos didáticos para a evolução das práticas pedagógicas, envolvendo a avaliação contínua dos métodos utilizados, bem como a discussão sobre novas abordagens e estratégias ativas que possam enriquecer o processo de ensino-aprendizagem em epidemiologia;
- Promover maior intercâmbio e troca de experiências entre os diferentes centros formadores em epidemiologia do país;
- Apoiar a formulação, a discussão e a disseminação de métodos e estratégias pedagógicas ativas para o ensino da epidemiologia, utilizando maior variedade de recursos tecnológicos e alinhando-se a competências e objetivos específicos de cada processo de aprendizagem;
- Promover a integração de metodologias ativas como estratégias pedagógicas para o ensino da epidemiologia nos componentes curriculares, garantindo que os processos educacionais sejam inclusivos, motivadores e transformadores;
- Induzir a oferta de programas de formação para docentes com foco no uso de tecnologias digitais de informação e comunicação, como parte de políticas institucionais;
- Incentivar estratégias, como mesas-redondas e oficinas de trabalho, sobre práticas docentes específicas para o ensino da epidemiologia na graduação.

#### Eixo 3: Articulação e Integração Ensino-Serviço-Pesquisa-Comunidade

Problemas sistematizados:

- Deficiência na formação integrada à prática nos serviços de saúde, evidenciando desconexão entre a teoria ensinada nos cursos e a realidade profissional;
- Limitação de currículos que não levam em consideração as particularidades de cada área de formação nem a necessidade de uma abordagem transversal entre as diferentes modalidades de ensino da epidemiologia;
- Resposta limitada diante das demandas dos serviços de saúde na rede de atenção do SUS na formação em epidemiologia nos cursos e programas de pós-graduação nas modalidades acadêmica e profissional, particularmente no que se remete às ações de vigilância;
- Deficiência na articulação e na interlocução entre instituições de ensino e serviços de saúde, comprometendo a conexão com a realidade prática e com profissionais da saúde no processo formativo e de educação permanente;
- Insuficiência de reflexão teórica e metodológica na análise de dados dos Sistemas de Informação em Saúde, assim como no uso das tecnologias digitais de informação e comunicação, como desafio significativo para a efetividade das intervenções em saúde pública. Essa lacuna tem limitado a elaboração de análises críticas e fundamentadas, reduzindo a capacidade de profissionais de saúde obterem informações relevantes para a tomada de decisão e para o planejamento estratégico;
- Uso limitado de *softwares* livres no ensino da epidemiologia, em todos os níveis de formação, como barreira significativa para a modernização e maior efetividade da educação nessa área;
- Necessidade de incorporação criteriosa e crítica da inteligência artificial (IA) no setor de saúde. Embora a IA tenha o potencial de transformar a atenção à saúde de pessoas doentes, a gestão de dados e a pesquisa em saúde, sua implementação deve ser acompanhada de uma análise cuidadosa de seus impactos éticos, sociais e operacionais;
- Avanço incipiente na incorporação de tecnologias ligadas à saúde digital nas instituições formadoras em oposição à demanda crescente da incorporação dessas tecnologias nos serviços de saúde, na vigilância e nos centros de referência;
- Deficiência na capacidade de tradução dos conhecimentos (popularização e disseminação) gerados nos diferentes processos formativos em epidemiologia para públicos específicos no país ou para a sociedade em geral.

Ações recomendadas para o enfrentamento dos problemas identificados:

- Promover a inclusão de componente curricular de vigilância em saúde nos cursos de graduação em saúde em geral e na pós-graduação em saúde coletiva, como foco no desenvolvimento do interesse e no conhecimento de discentes sobre práticas eficazes de enfrentamento de problemas de saúde no país;
- Estimular espaços de práticas nos serviços de saúde, sobretudo na vigilância em saúde, nos cursos de graduação e pós-graduação do campo da saúde coletiva;
- Promover estágios extracurriculares para cursos de graduação que colaborem com a aproximação entre discentes e a prática da epidemiologia no âmbito dos serviços de saúde no SUS e dos territórios de comunidades;
- Promover iniciativas que favoreçam a aproximação e a integração de gestores(as) com profissionais de saúde nos processos formativos e de educação permanente;
- Incorporar as necessidades do serviço de saúde no desenvolvimento de produção bibliográfica e técnico-tecnológica das diferentes modalidades de cursos, contribuindo para que a formação acadêmica atenda às demandas reais do SUS;
- Estimular estratégias de educação permanente em epidemiologia voltadas para profissionais de saúde;
- Promover espaços de reflexão teórica e metodológica para análise de Sistemas de Informação em Saúde e para uso de tecnologias digitais de informação e comunicação;
- Incentivar a criação de um portal de acesso público para a disponibilização de *softwares* livres voltados para a epidemiologia;
- Estimular a formação de discentes, gestores(as) e pesquisadores(as) com habilidades para a aplicação de métodos e técnicas de tradução dos conhecimentos (popularização e disseminação) produzidos na sociedade.

### Área temática II: A Pesquisa em Epidemiologia

#### Eixo 1: Desigualdades Sociais e Iniquidades em Saúde

Problemas sistematizados:

- Insuficiência de pesquisas que integrem o modelo econômico em suas abordagens teóricas, propostas metodológicas e avaliações de impactos na saúde;
- Reduzido número de estudos e escasso conhecimento produzido sobre populações vulnerabilizadas, como as que vivem em situação de rua, em privação de liberdade, povos indígenas, populações rurais/ribeirinhas, populações do campo, da floresta e das águas, população LGBTQIAPN+, quilombolas e pessoas com deficiência.

Ações recomendadas para o enfrentamento dos problemas identificados:

- Reforçar a importância de que as pesquisas enfatizem as origens e a produção das desigualdades socioeconômicas originadas no coração do sistema capitalista e que são fortes produtoras de iniquidades em saúde. Considerando o estado atual do processo de concentração da renda e de implementação de políticas neoliberais, reforça-se a necessidade de ampliar o número de pesquisas para monitorar o impacto desses processos na pobreza, nos indicadores de saúde e na magnitude das desigualdades sociais em saúde;
- Além do impacto desse modelo econômico, recomenda-se que as condições de vida e saúde da grande maioria da população brasileira, desprivilegiada, excluída e oprimida, sejam foco prioritário de investigações. Nessas populações, potencializar maior destaque à população negra e feminina, historicamente discriminada, desvalorizada e excluída;
- Estimular componentes curriculares críticos e discussões periódicas sobre os impactos na saúde de políticas econômicas e sociais, processo produtivo e desigualdades estruturais, de modo a ampliar pesquisas baseadas em sólidos modelos teóricos que envolvam determinantes sociais em saúde, agregando as perspectivas dos diversos núcleos disciplinares da saúde coletiva;
- Fomentar o desenvolvimento de indicadores específicos para mensuração da interseccionalidade e da complexidade que envolvam transdisciplinaridade e pluralidade de abordagens metodológicas, diante dos desafios operacionais da incorporação das desigualdades e iniquidades em saúde aos modelos de análise;
- Incentivar pesquisas que promovam a reflexão crítica sobre as diferentes teorias sociológicas aplicadas a modelos de determinação social do processo saúde-doença e das condições de saúde;
- Promover o uso plural, crítico e criativo de métodos de investigação em estudos sobre desigualdades sociais em saúde, com o envolvimento das comunidades estudadas no processo de pesquisa, a adoção de indicadores adequados para medir eventos de saúde em populações vulnerabilizadas e que contemplem a interseccionalidade. Isso pode incluir a formação de parcerias com organizações comunitárias, a realização de consultas prévias e o respeito às culturas e aos conhecimentos tradicionais;
- Incentivar políticas públicas de fomento à realização de estudos — como editais, bolsas de estudo, entre outras — e a divulgação de resultados de pesquisas com enfoque nas desigualdades sociais em saúde no Brasil. Isso inclui as desigualdades regionais com foco no impacto das políticas macroestruturais e do processo de concentração da renda na saúde e na qualidade de vida das populações vulnerabilizadas;
- Ampliar a articulação entre grupos de pesquisa e programas de pós-graduação para estudos colaborativos sobre iniquidades em saúde, compartilhando conhecimentos, iniciativas, instrumentos e técnicas para diversificar e maximizar estudos na área. As desigualdades inter- e intrarregionais do país devem ser consideradas nessas iniciativas mediante, por exemplo, a inclusão de eventos científicos com discussões periódicas sobre o tema;
- Demandar o desenvolvimento de políticas públicas e iniciativas acadêmicas, tais como a abertura de editais de financiamento específicos para o desenvolvimento de pesquisas estratégicas com diferentes populações em contextos de maior vulnerabilidade;
- Ampliar a diversidade nos conselhos editoriais e a proposição de números temáticos sobre subgrupos populacionais em situação de maior vulnerabilidade em revistas científicas do campo da saúde coletiva;
- Estimular a oferta de disciplinas e a realização de seminários que incentivem a pesquisa sobre subgrupos populacionais em situação de maior vulnerabilidade;
- Incentivar e apoiar metodologias de pesquisa participativa que envolvam as comunidades estudadas no processo de pesquisa em seus territórios, por meio de parcerias com organizações comunitárias e a realização de consultas prévias, fortalecendo o respeito às culturas e aos conhecimentos tradicionais;
- Induzir cursos e oficinas de trabalho especializados em pesquisa com grupos populacionais vulnerabilizados nos congressos de epidemiologia e instigar maior diversidade e integração entre os grupos temáticos da Abrasco que tenham interfaces com o tema;
- Reforçar e incentivar que as pesquisas relacionadas a populações em situação de maior vulnerabilidade tenham como referencial teórico os direitos humanos e sociais, ressaltando-se as pessoas com deficiências, com destaque às que têm dupla carga de discriminação, pois, além de serem restringidas em seus direitos fundamentais, são consideradas pelo modelo econômico neoliberal menos importantes para o sistema capitalista, por, supostamente, serem menos produtivas.

#### Eixo 2: Financiamento para Pesquisas e Instituições de Pesquisa

Problema sistematizado:

- Insuficiência de políticas robustas e estáveis de financiamento científico, comprometendo a democratização, o fortalecimento, a expansão e a internacionalização de grupos de pesquisa brasileiros.

Ações recomendadas para o enfrentamento do problema identificado:

- Debater com agências de fomento e órgãos governamentais municipais, estaduais e federais a necessidade da criação de políticas mais robustas e estáveis de financiamento para pesquisas epidemiológicas, bem como fortalecer iniciativas de fomento por meio da colaboração entre países do Sul Global;
- Estimular o fortalecimento de políticas de financiamento que apoiem jovens doutores(as), prevenindo a evasão da carreira científica e a fuga de talentos para o exterior. Isso inclui a disponibilidade de bolsas de fixação ou de pós-doutorado para atuação em redes colaborativas, além da abertura de mais concursos públicos, para a composição de quadros em universidades, institutos e fundações, acompanhados de adequada infraestrutura para garantia das condições para conformação de grupos de pesquisa;
- Mobilizar a comunidade científica a produzir evidências sobre o impacto do investimento público em ciência e tecnologia no desenvolvimento do país para o avanço do conhecimento e na qualificação de políticas públicas, de modo a fundamentar as demandas às agências de fomento e governamentais por recursos de pesquisa contínuos e vigorosos;
- Estimular que agências de fomento considerem as desigualdades regionais na abertura de editais de pesquisa, agindo com equidade e contemplando necessidades específicas de cada região do país e das populações mais vulnerabilizadas;
- Proporcionar oportunidades de formação para que jovens pesquisadores(as) possam elaborar propostas competitivas e gerenciar os recursos de maneira eficaz, ampliando a capacidade de captação de recursos nacionais e externos para fomento da pesquisa epidemiológica no país;
- Demandar que a seleção de projetos e grupos de pesquisa a serem financiados com recursos públicos seja prioritariamente realizada por meio de editais públicos amplamente divulgados e que as contratações diretas de grupos de pesquisa sejam restritas a situações específicas, tais como aquelas relacionadas a emergências em saúde pública;
- Promover a discussão sobre financiamentos para repositórios de dados confiáveis que atendam à Lei Geral de Proteção de Dados Pessoais e que estejam disponíveis para uso de pesquisadores(as) e gestores(as) da saúde.

#### Eixo 3: Teoria, Método e Ética em Pesquisa

Problemas sistematizados:

- Lacuna teórica e limitações metodológicas em pesquisas epidemiológicas, restringindo o desenvolvimento de pesquisas com impactos sociais significativos;
- Insuficiência de abordagens multidimensionais e interdisciplinares que permitam explorar a complexidade de problemas e contextos da saúde;
- Uso de dados secundários em saúde com pouca reflexão e aprofundamento sobre suas potencialidades e limitações para a análise epidemiológica;
- Limitado acesso a bases de dados governamentais para fins de pesquisa;
- Falta de repositórios confiáveis e seguros para guarda de dados resultantes de pesquisas epidemiológicas com vistas à implementação de estratégias de ciência aberta;
- Inexistência de debate sobre adequação de questões éticas em pesquisas populacionais.

Ações recomendadas para o enfrentamento dos problemas identificados:

- Valorizar a formação crítica de pesquisadores(as), fortalecendo com eles(as) a necessidade de estudos com robusta reflexão teórica e a busca por métodos de pesquisa que permitam avanços significativos no conhecimento e produzam evidências sólidas para subsidiar o enfrentamento de problemas concretos de saúde;
- Estimular a criação e o fortalecimento de redes de pesquisa visando à difusão de teorias e métodos, incorporando programas de pós-graduação e grupos de pesquisa de diferentes regiões e graus de maturidade;
- Desenvolver ações de formação continuada para docentes e pesquisadores(as), promovendo o fortalecimento e a inovação em métodos e teorias da pesquisa epidemiológica;
- Incentivar o debate e a implementação de iniciativas que reduzam a valorização do produtivismo científico, promovendo apoio a pesquisas com relevância científica e social e com aprofundamento teórico e metodológico e à adoção de boas práticas editoriais e de publicação científica;
- Estimular a maior integração entre grupos de pesquisa e programas de pós-graduação de diferentes áreas e distintas vocações e o desenvolvimento de editais específicos que exijam ou incentivem a formação de equipes de pesquisa com *expertise* diversa, incluindo áreas como sociologia, antropologia, psicologia, saúde pública, economia e estudos culturais;
- Compor grupos de trabalho para identificar estratégias aplicáveis à saúde coletiva e à pesquisa epidemiológica alinhadas à estratégia de saúde digital para o Brasil que estimulem a pesquisa, a inovação e o desenvolvimento de soluções em IA, com ênfase ao desenvolvimento de soluções para problemas relevantes à saúde da população brasileira;
- Estimular o financiamento de estudos que desenvolvam ferramentas da pesquisa epidemiológica e avaliem sua implementação nos serviços de saúde;
- Incentivar a cooperação internacional para compartilhamento de novas tecnologias, boas práticas e experiências bem-sucedidas em saúde coletiva que tenham sido desenvolvidas em outros contextos nacionais;
- Promover eventos e cursos que incentivem e instrumentalizem pesquisadores(as) no uso qualificado e criativo dos dados secundários de saúde. Incentivar que cursos de graduação, pós-graduação e técnicos discutam a importância da análise de dados dos Sistemas de Informação em Saúde e orientem análises que promovam avanços no conhecimento e na aplicação pelos serviços;
- Apoiar a educação permanente das equipes de vigilância e demais profissionais de saúde para coleta e entrada de dados nos Sistemas de Informação, visando aumentar sua qualidade, sua completude e seu uso qualificado pelos serviços de saúde;
- Promover a colaboração entre profissionais atuantes em epidemiologia, estatística, ciências humanas e sociais e gestão de dados e outros coletivos profissionais para que a análise de dados secundários seja feita com múltiplas perspectivas e distintos métodos e técnicas;
- Demandar o aprimoramento contínuo das bases de dados existentes no Brasil, especialmente no que se refere à ampliação de cobertura, atualização, qualificação das informações e padronização de variáveis para facilitar a integração entre os Sistemas de Informação do Ministério da Saúde e de outros setores governamentais;
- Dialogar com o Ministério da Saúde e outros órgãos governamentais diante da necessidade de acesso aberto, democrático e transparente a dados secundários de interesse epidemiológico em toda a sua abrangência, respeitando-se a Lei Geral de Proteção de Dados Pessoais;
- Criar ambientes de diálogo entre pesquisadores(as), controle social, operadores(as) e controladores(as) de bases de dados para, adequada e uniformemente, interpretar a Lei Geral de Proteção de Dados Pessoais, de modo a garantir a utilização ética dos dados, ao mesmo tempo que se viabilize o acesso a eles;
- Promover debates sobre financiamentos e desenvolvimento de repositórios nacionais de dados com credibilidade e segurança, que atendam à Lei Geral de Proteção de Dados Pessoais e que estejam disponíveis para o uso de pesquisadores(as) e gestores(as) da saúde;
- Desenvolver materiais instrucionais e debates mais abertos com pesquisadores(as) e gestores(as) sobre a importância da ciência aberta, a gestão segura de dados e o uso adequado de repositórios;
- Fomentar debates sobre iniciativas inovadoras para o aprimoramento de normas e fluxos de procedimentos éticos, adequando-os aos diferentes tipos de estudos populacionais observacionais na epidemiologia.

### Área Temática III: a Epidemiologia em Políticas, Programas e Serviços de Saúde

#### Eixo 1: Sistemas Nacionais de Informação e Práticas Epidemiológicas: Análise de Situação de Saúde Segundo Condições de Vida

Problemas sistematizados:

- Limitação da interoperabilidade dos múltiplos Sistemas de Informação em Saúde e tecnologias utilizados no SUS e de Sistemas de Informação de outros setores ou instituições governamentais para análises epidemiológicas. As dificuldades são demarcadas por fragmentação operacional, restrições técnicas e/ou semânticas e inexistência de código identificador único e de padronização do perfil identificador do cidadão, reduzindo as possibilidades de acesso e a intercomunicabilidade na utilização efetiva dos dados disponíveis;
- Insuficiência de disponibilidade e qualidade dos dados, bem como de acesso e utilização dos Sistemas de Informação em Saúde para alcance de dimensões epidemiológicas de doenças/agravos/eventos e seus determinantes, considerando particularmente populações mais vulnerabilizadas nos diferentes contextos sociais do país;
- Restrição do acesso a dados da vigilância em saúde e das Redes de Atenção à Saúde, incluindo aqueles vinculados a planos e serviços de saúde privados, como clínicas, hospitais, laboratórios, farmácias e unidades de vacinação;
- Fragilidade das ações de consolidação de dados no SUS, de análises epidemiológicas e de divulgação de informações, limitando a retroalimentação oportuna e qualificada de informações críticas para tomada de decisão e divulgação para a sociedade;
- Insuficiência de investimentos para garantia de infraestrutura necessária e de pessoal qualificado para gestão em saúde com foco em manutenção, atualização e operacionalização de Sistemas de Informação em Saúde em diferentes âmbitos de gestão do SUS.

Ações recomendadas para o enfrentamento dos problemas identificados:

- Fortalecer, no âmbito da Abrasco, a estruturação de espaços estratégicos para ampliação da participação técnico-científica em instâncias de tomada de decisão em saúde no Estado brasileiro legitimados pelo reconhecimento de grupos de trabalho temáticos já existentes ou a serem constituídos na Comissão de Epidemiologia;
- Advogar que a propriedade e a gestão de dados oriundos de Sistemas Nacionais de Informação em Saúde sejam de responsabilidade exclusiva do setor público do país em *datacenters*, em território nacional e indelegáveis, fundamentadas em princípios éticos, democráticos e soberanos;
- Defender um plano de investimentos no país para a consolidação de ações estratégicas para acesso à informação em saúde, bem como disponibilidade e qualidade, em nível federal e em estados e municípios, pela atuação de espaços legítimos para tomada de decisão e com critérios para aplicação responsável da Lei Geral de Proteção de Dados Pessoais diante das prioridades do país;
- Propor que os Sistemas Nacionais de Informação considerem a criação de um código identificador único para cada cidadão(ã) e a padronização do perfil identificador do cidadão — conjunto mínimo de dados (inserção qualificada de campos de nome social, identidade de gênero, orientação sexual, raça, etnia, pessoas com deficiência, entre outros) —, salvaguardando princípios da Lei Geral de Proteção de Dados Pessoais;
- Defender o acesso oportuno a dados do sistema privado de saúde, considerando a multiplicidade de instituições, serviços e planos de saúde, com foco principal nas ações de vigilância, mas também em pesquisas estratégicas em saúde para o país, como no contexto de emergências em saúde pública;
- Indicar ações que contribuam para a sustentabilidade dos Sistemas Nacionais de Informação por meio da Rede Nacional de Dados em Saúde, com garantia de interoperabilidade técnica, operacional e semântica de codificações, de modo integrado e disponível a outros setores, como educação, contextos sociais e ambiente;
- Contribuir para a ampliação da produção de conhecimento técnico-científico, com atenção à necessária equidade perante as desigualdades regionais e condições determinadas socialmente, com ênfase em populações e territórios mais vulnerabilizados, fundamentado pela consolidação e pela análise de bases de dados dos Sistemas Nacionais de Informação e por suas interfaces intersetoriais;
- Recomendar a adoção e o aperfeiçoamento de diretrizes e políticas estratégicas para acesso a dados de saúde, partindo do princípio elementar do direito à informação, tanto de dados oriundos da vigilância em saúde quanto das Redes de Atenção à Saúde no SUS.

#### Eixo 2: Formação de Pessoas

Problemas sistematizados:

- Desafios na formação durante a graduação e a pós-graduação para ampliar a compreensão e a utilização da epidemiologia no lidar com infodemia, desinfodemia, negacionismo e desinformação com base na visão crítica das evidências produzidas por estudos;
- Desafios para fomento de processos formativos ainda mais inclusivos, que levem em consideração a diversidade humana, como orientação sexual, identidade de gênero, raça, etnia, classe social e pessoas com deficiência;
- Inexistência de carreira e contratos estáveis para profissionais sanitaristas com formação em epidemiologia que respondam às necessidades das políticas públicas do SUS para tomada de decisão;
- Insuficiência de investimentos na Política Nacional de Educação Permanente em Saúde (PNEPS) para fomento de iniciativas promotoras de análises epidemiológicas, avaliação de impacto, uso de relatórios de situação de saúde, comunicação em saúde e análise de todo o potencial das bases de dados disponíveis.

Ações recomendadas para o enfrentamento dos problemas identificados:

- Potencializar estratégias de formação, educação permanente, monitoramento, avaliação, supervisão, informação e comunicação no SUS, com foco na qualificação da gestão e na adoção de práticas epidemiológicas qualificadas. Essas estratégias devem subsidiar a construção de indicadores relevantes e promover a análise consistente da situação de saúde para tomada de decisão. Requerem qualificar a formação de trabalhadores e trabalhadoras em cursos de saúde em nível técnico, na graduação e na pós-graduação;
- Ampliar ações de advocacia e de constituição de comitês técnico-científicos para implementação da carreira de sanitarista no SUS conforme as normativas legais vigentes do Congresso Nacional e com forte base formativa em epidemiologia, nas redes de atenção e vigilância, para fortalecimento da gestão em saúde, considerando competências, responsabilidades e critérios para cada nível nas ações de gestão, atenção, vigilância, prevenção, controle, pesquisa e ensino;
- Contribuir para a implementação da PNEPS com base em iniciativas que estimulem a incorporação de novas estratégias de uso do raciocínio e do método epidemiológico de base territorial nas ações de vigilância para ampliar a capacidade de execução de análises epidemiológicas prioritárias, incluindo análise situacional, monitoramento e avaliação de processos, além de avaliação de efetividade, de custo-benefício e de impacto das ações em saúde;
- Contribuir para a adoção de práticas formativas nos cursos técnicos de saúde (nível médio), de graduação e de pós-graduação (*lato sensu*, com destaque às residências, e *stricto sensu*, com destaque a cursos de mestrado e doutorado na modalidade profissional) e na educação permanente de trabalhadores(as) da saúde que levem em consideração a diversidade humana, como orientação sexual, identidade de gênero, raça, etnia, classe social e pessoas com deficiência, tanto por meio de políticas afirmativas quanto por outras estratégias para superar as persistentes desigualdades sociais no país;
- Propor o fortalecimento de estratégias formativas inovadoras que incorporem conhecimentos sobre novas ferramentas para sistematização e análise de dados, bem como a tradução do conhecimento técnico-científico (popularização e disseminação) em bases sólidas de informação e comunicação em saúde no país, com foco em ampliar a capacidade nacional de resposta a situações de infodemia, desinfodemia, negacionismo e desinformação.

#### Eixo 3: Vigilância em Saúde

Problemas sistematizados:

- Insuficiência de articulação/integração entre atenção e vigilância em saúde no SUS, considerando todos os pontos e instâncias das Redes de Atenção à Saúde por ciclos de vida, doenças, agravos, eventos e determinantes sociais;
- Limitação da efetividade dos componentes da vigilância em saúde para populações mais vulnerabilizadas, como povos indígenas, quilombolas, assentadas, em situação de rua, migrantes, refugiadas e apátridas, privadas de liberdade, LGBTQIAPN+, ciganas e circenses;
- Insuficiência na incorporação de novas estratégias para vigilância, tais como vigilância popular e participativa, vigilância genômica, identificação oportuna de novos patógenos/mutantes, vigilância preditiva e uso de mídias sociais para captura de eventos;
- Limitação da incorporação de diferentes dimensões do território e de contextos locais, ampliando desafios do trabalho com determinantes sociais e fatores de risco;
- Limitação da disponibilidade de informação simplificada para a gestão em saúde e a sociedade, com vistas à tradução dos conhecimentos (popularização e disseminação) para plena comunicação e participação em saúde.

Ações recomendadas para o enfrentamento dos problemas identificados:

- Estimular a implementação de pesquisas prioritárias em diferentes realidades do SUS, com forte participação social e foco na qualificação das ações da vigilância em saúde com interfaces a Redes de Atenção à Saúde, valorizando perspectivas transdisciplinares e interepistêmicas de enfrentamento das desigualdades sociais e iniquidades;
- Contribuir para o desenvolvimento e a ampla comunicação de indicadores estratégicos da vigilância em saúde de base territorial em suas diferentes dimensões, incluindo aqueles que tenham multicritérios com integração de perspectivas de determinação social para definição de áreas prioritárias, com foco no desenvolvimento de ações de enfrentamento a diferentes desafios e problemas reconhecidos no SUS;
- Contribuir para a adoção de evidências geradas pelos Sistemas Nacionais de Informação para tomada de decisões no SUS pautadas em métodos e técnicas da epidemiologia, integrados a estratégias de base territorial, como vigilância popular, cidadã e participativa, vigilância genômica, identificação oportuna de novos patógenos/mutantes, vigilância preditiva, utilização de mídias sociais para captura de rumores e eventos e novas tecnologias;
- Cooperar para a implementação do uso de métodos e técnicas da epidemiologia para análise de desigualdades sociais e iniquidades no SUS, bem como da articulação transdisciplinar, interepistêmica e intersetorial necessárias em iniciativas governamentais que abordem essa temática na perspectiva da saúde coletiva;
- Estabelecer prioridades para qualificação, estruturação e organização das ações da rede nacional de laboratórios de saúde pública, como parte integrante da vigilância em saúde de base territorial;
- Defender a ampliação do financiamento de políticas e programas que envolvam ações estratégicas do complexo econômico e industrial da saúde para superação de barreiras do acesso à saúde no país como direito humano;
- Defender a efetiva implementação da Política Nacional de Vigilância em Saúde e da Política Nacional de Saúde do Trabalhador e da Trabalhadora, que garantam a integração com a Política Nacional de Atenção Básica mediante agendas de trabalho comuns e com operacionalização intersetorial.

#### Eixo 4: Avaliação de Programas, Serviços e Intervenções em Saúde

Problemas sistematizados:

- Fragilidade de iniciativas para monitoramento e avaliação epidemiológica do impacto de modelos da vigilância em saúde em contextos atuais do país como fonte de evidências para a sua efetiva reformulação;
- Fragilidade de políticas, programas e ações integradas voltados para a estruturação e organização de rede de laboratórios (em saúde pública e privada), como apoio à estruturação, ao monitoramento e à avaliação das ações de vigilância em saúde;
- Insuficiência no financiamento para desenvolvimento de ações de monitoramento e avaliação epidemiológica;
- Limitação do processo de indução para que programas de pós-graduação profissionais (mestrados e doutorados) incorporem o desenvolvimento de estudos epidemiológicos com temáticas prioritárias para o país em implementação e em monitoramento e avaliação de programas, serviços e intervenções em saúde;
- Limitação do alcance das perspectivas trazidas pela epidemiologia para respostas a demandas e prioridades nacionais de modo articulado com ações do Ministério da Saúde.

Ações recomendadas para o enfrentamento dos problemas identificados:

- Ampliar e fortalecer no país os canais de divulgação da produção bibliográfica da epidemiologia, como revistas, eventos técnico-científicos, *websites*, plataformas e repositórios, para divulgar ações exitosas da epidemiologia desenvolvidas em diferentes territórios e espaços de gestão do SUS, priorizando perspectivas fundamentadas na determinação social e o diálogo com a sociedade brasileira;
- Avançar em desenvolvimento, qualificação, valorização e divulgação de produções técnicas e tecnológicas vinculadas a análises situacionais, visando propor soluções de problemas inerentes à vigilância e à atenção que incluam estratégias formativas nos cursos técnicos de saúde (nível médio), de graduação e de pós-graduação (*lato sensu*, com destaque às residências, e *stricto sensu*, em especial cursos de mestrado e doutorado na modalidade profissional), além da educação permanente de trabalhadores(as) nos territórios e serviços do SUS;
- Propor o desenvolvimento de iniciativas, para qualificação das ações de monitoramento e avaliação, com foco em um sistema descentralizado e integrado de dados e informações da vigilância e das Redes de Atenção à Saúde. Tais iniciativas devem valorizar a consolidação por sua construção democrática, respeitar as diversidades regionais e populacionais e reconhecer experiências bem-sucedidas, como as apresentadas na Mostra Nacional de Experiências Bem-Sucedidas em Epidemiologia, Prevenção e Controle de Doenças;
- Defender a ampliação da infraestrutura de tecnologia de informação na rede de atenção e vigilância do SUS, com adequação de equipamentos e maior cobertura e qualidade da internet para implementação de fluxos operacionais qualificados e seguros, tendo como base a disponibilidade de pessoal qualificado;
- Defender a ampliação do financiamento destinado a ações formativas estratégicas para maior capacidade de planejamento, monitoramento e avaliação pautadas no método epidemiológico, com foco na qualificação da tomada de decisão nos diferentes níveis de gestão do SUS, com envolvimento de instituições de ensino e pesquisa na formação de pessoas por intermédio de estratégias inovadoras, como o Programa de Educação pelo Trabalho para Saúde, para vigilância em saúde, e os cursos de pós-graduação na modalidade profissional;
- Contribuir com o desenvolvimento de métodos e técnicas de monitoramento e avaliação participativa das ações de produção de informação e incorporação de novas tecnologias em todos os níveis de atenção, vigilância e gestão do SUS.

#### Eixo 5: Inserção da Epidemiologia nas Políticas Intra e Intersetoriais

Problemas sistematizados:

- Dificuldade de implementação das perspectivas e abordagens da epidemiologia sobre iniquidades em saúde, bem como da articulação inter e transdisciplinar entre as três formações discursivas do campo da saúde coletiva;
- Heterogeneidade e considerável complexidade do uso da epidemiologia nos diversos níveis de gestão do SUS e entre eles para tomada de decisão baseada em evidências em políticas públicas;
- Insuficiente valorização e divulgação das ações exitosas da epidemiologia desenvolvidas em territórios e espaços de gestão do SUS;
- Insuficiência de iniciativas de colaboração em rede de programas de pós-graduação (na área da saúde coletiva), pesquisadores(as), instituições e serviços de saúde com vistas à ampliação da integração extensão-ensino-serviço e comunidade como política de Estado, e não apenas de governo.

Ações recomendadas para o enfrentamento dos problemas identificados:

- Contribuir para a ampliação da mobilização e do engajamento social com ampla participação da sociedade civil, respeitando a garantia de representatividade e diversidade, via lideranças, movimentos e organizações sociais, nos espaços sociopolíticos decisórios de atuação da Abrasco, potencializando ações estratégicas exitosas da epidemiologia desenvolvidas em diferentes territórios e espaços de gestão do SUS;
- Contribuir para a construção de espaços de maior integração entre comitês temáticos e grupos de trabalho da Abrasco, com vistas a reconhecer e ampliar iniciativas estratégicas de formação e de comunicação em rede de cursos de graduação e de programas de pós-graduação com foco na solidariedade e colaboração. Essas iniciativas devem integrar, traduzir e divulgar prioridades na saúde da sociedade brasileira, alcançadas por meio de ações de vigilância em saúde, incluindo práticas epidemiológicas. Além disso, devem considerar a ampliação da integração extensão-ensino-serviço e comunidade e a valorização de espaços democráticos, participativos e legítimos no país;
- Contribuir para a ampliação de estratégias de mobilização e participação de lideranças, representações de Conselhos de Saúde e de movimentos sociais pela educação, visando ao fortalecimento da Rede Nacional de Formação em Vigilância em Saúde no SUS, cidadã, democrática e participativa.

## Figures and Tables

**Figura 1:** Número de participantes das oficinas segundo instituição de origem.

Fiocruz: Fundação Oswaldo Cruz; Opas: Organização Pan-Americana da Saúde; SVSA: Secretaria de Vigilância em Saúde e Ambiente; UFAC: Universidade Federal do Acre; UFBA: Universidade Federal da Bahia; UFC: Universidade Federal do Ceará; UFPE: Universidade Federal de Pernambuco; UFMA: Universidade Federal do Maranhão; UFRB: Universidade Federal do Recôncavo Baiano; UEFS: Universidade Estadual de Feira de Santana; UNB: Universidade de Brasília; UFMT: Universidade Federal do Mato Grosso; UERJ: Universidade do Estado do Rio de Janeiro; UFRJ: Universidade Federal do Rio de Janeiro; UFF: Universidade Federal Fluminense; USP: Universidade de São Paulo; Unicamp: Universidade Estadual de Campinas; UFVJM: Universidade Federal dos Vales do Jequitinhonha e Mucuri; UFSJ: Universidade Federal de São João del-Rei; UFSC: Universidade Federal de Santa Catarina; UFRGS: Universidade Federal do Rio Grande do Sul; UFPEl: Universidade Federal de Pelotas.

## Data Availability

Todo o conjunto de dados que dá suporte aos resultados deste estudo está disponível mediante solicitação à autora correspondente, Tânia Maria de Araújo. O conjunto de dados não está publicamente disponível por causa da presença de informações sensíveis com potencial de identificação dos/das participantes. A coleta desses dados ocorreu em contextos colaborativos e requer tratamento ético e responsável. Assim, o acesso é restrito para garantir a privacidade dos envolvidos e respeitar os princípios de proteção de dados pessoais. Portanto, o acesso é restrito em razão de exigências éticas relacionadas à confidencialidade dos participantes.

## ANEXO 1.

### Informações adicionais sobre o processo de elaboração do V Plano Diretor para o Desenvolvimento da Epidemiologia no Brasil (2025–2029) e o perfil dos(as) participantes envolvidos(as).

O processo de elaboração do V Plano Diretor para o Desenvolvimento da Epidemiologia no Brasil (2025–2029) foi estruturado em duas fases:

- Fase 1: diagnóstico dos principais problemas avaliados em três áreas temáticas: I. Formação; II. Pesquisa; III. A Epidemiologia nas Políticas, Programas e Serviços de Saúde;
- Fase 2: proposição de ações e estratégias de enfrentamento dos problemas prioritários definidos na fase 1.

Em cada fase, foi conduzido um inquérito eletrônico, elaborados relatórios técnicos preliminares e realizada uma oficina presencial para discussão e deliberação. O primeiro inquérito objetivou identificar os problemas prioritários em cada eixo, servindo de base aos relatórios que orientaram a primeira oficina.

Em Brasília, nos dias 17 e 18 de agosto de 2023, foi realizada a **I Oficina** para a elaboração do **V Plano Diretor para o Desenvolvimento da Epidemiologia no Brasil (2025–2029)**, executando, assim, a **fase 1** do processo de elaboração. O diagnóstico dos problemas foi consolidado em relatório técnico, oriundo das discussões e relatoria dos grupos nas três áreas temáticas mencionadas anteriormente. Na Figura 1 estão descritas as características gerais do grupo participante das oficinas.

Com base nas informações do relatório técnico da **fase 1** (definição dos problemas prioritários), foi desenhado um inquérito para levantamento de sugestões de ações para superação dos problemas identificados. Assim, foi encaminhado aos/às participantes da I Oficina um formulário eletrônico para registro de sugestões de estratégias de enfrentamento aos problemas.

Como preparação para a II Oficina, foi elaborado um relatório técnico preliminar com sugestões coletadas por meio do inquérito eletrônico. O relatório reuniu contribuições destinadas a orientar as discussões e definir ações para o desenvolvimento da epidemiologia nos cinco anos seguintes, as quais foram discutidas e aprovadas na II Oficina.

A II Oficina ocorreu nos dias 12 e 13 de setembro de 2024, no Rio de Janeiro, com o objetivo de execução da **Fase 2**, voltada à discussão e definição das propostas de ação.

O processo de elaboração do Plano Diretor envolveu um amplo conjunto de epidemiologistas de todas as regiões do país. A Tabela 1 apresenta o perfil dos/as participantes envolvidos(as) em pelo menos uma das etapas realizadas, incluindo os inquéritos eletrônicos e as oficinas.

**Tabela 1:** Perfil dos(as) participantes nas diferentes etapas do processo de elaboração do V Plano Diretor para o Desenvolvimento da Epidemiologia no Brasil (2025–2029). Brasil, 2023–2024.

Características		%
Região		
	Norte	4,5
	Nordeste	20,9
	Centro-Oeste	18,2
	Sudeste	40,9
	Sul	15,5
**Características gerais dos/as informantes**		
Gênero		
	Mulher cisgênero	60,7
	Homem cisgênero	36,4
	Travesti	1,0
	Prefiro não informar	1,9
Faixa etária (anos)*		
	Até 41	26,7
	42–50	22,9
	51–64	25,7
	65 ou mais	24,8
Raça/cor		
	Branca	77,5
	Preta e parda	21,5
	Amarela (asiática)	1,0
Graduação**		
	Medicina	28,7
	Nutrição	13,9
	Odontologia	7,4
	Enfermagem	6,5
	Fisioterapia	6,5
	Farmácia	5,6
	Educação Física	4,6
	Estatística	4,6
Local de trabalho principal		
	Universidade pública	66,6
	Gestão pública em saúde	21,2
	Centro de pesquisa	9,1
	Universidade particular	3,1
Tempo que trabalha como epidemiologista (anos)		
	Até 10	25,7
	11–15	19,0
	16–25	26,7
	26 ou mais	28,6
Campo de atuação atual***		
	Atividade acadêmica/ensino	78,8
	Atividade de pesquisa	84,8
	Atividade em serviços de saúde	36,4

* Média de idade: 51,2 anos; mediana: 51,0 anos; idade mínima: 29 anos; idade máxima: 79 anos;

** outras graduações referidas: Saúde Coletiva (n=3; 2,8%), Biomedicina (n= 3; 2,8%), Ciências Biológicas (n=3; 2,8%), Fonoaudiologia (n=3; 2,8%); Ciências Sociais (n=1; 1,9%), Engenharia (n=1; 1,9%), Geografia (n=1; 1,9%), Matemática (n=1; 1,9%), Psicologia (n=1; 1,9%), Serviço Social (n=1; 1,9%);

*** variável com múltiplas respostas.

# Translation: V Plan Director para el Desarrollo de la Epidemiología en Brasil (2025–2029)

**DOI:** 10.1590/1980-549720250001.supl.1.3

**Authors:** Tânia Maria de Araújo, Enirtes Caetano Prates Melo, Claudia Leite de Moraes, Laio Magno, Ana Paula Muraro, Maria de Jesus Mendes da Fonseca, Alberto Novaes Ramos, Luana Giatti Gonçalves, Margareth Guimarães Lima, Ligia Regina de Oliveira, Moisés Goldbaum, Joilda Silva Nery, Maria Rita Donalisio, Maria da Glória Lima Cruz Teixeira, Katia Vergetti Bloch, Antonio Fernando Boing, Guilherme Loureiro Werneck, *Comisión de Elaboración del Plan Director, Área Temática I: Formación en Epidemiología, Amanda de Moura Souza, Edna Massae Yokoo, Elisabeth Carmen Duarte, Emanuele Souza Marques, Janaína dos Santos Motta, João Roberto Cavalcante Sampaio, Juan José Cortez Escalante, Katia Crestine Poças, Lívia Teixeira Souza Maia, Michael Eduardo Reichenheim, Naomar Monteiro de Almeida-Filho, Paola Barbosa Marchesini, Rodrigo Citton Padilha dos Reis, Sérgio Viana Peixoto, Área Temática II: La Investigación en Epidemiología, Ana Paula Nogueira Nunes, Andréia Moreira de Andrade, Antônio Augusto Moura da Silva, Carolina Geralda Alves, Felipe Guimarães Tavares, Fernando José Herkrath, Isis Polianna Silva Ferreira de Carvalho, Jesem Douglas Yamall Orellana, Ligia Regina Franco Sansigolo Kerr, Maria Amélia de Sousa Mascena Veras, Maria Cynthia Braga, Maria do Carmo Leal, Marilisa Berti de Azevedo Barros, Mauricio Lima Barreto, Paloma de Sousa Pinho, Paulo Rossi Menezes, Sandhi Maria Barreto, Vinícius Silva Belo, Wildo Navegantes de Araújo, Área Temática: Epidemiología en Políticas, Programas y Servicios de Salud, Alícia Krüger, Amarílis Bahia Bezerra, Bárbara Campos Silva Valente, Carmen Silvia Bruniera Domingues, Claudio Maierovitch Pessanha Henriques, Ethel Leonor Noia Maciel, Fatima Sonally Sousa Gondim, Gerusa Belo Gibson dos Santos, Gulnar Azevedo e Silva, Gustavo Laine Araújo de Oliveira, Heleno Rodrigues Corrêa, Luciana de Carvalho Penna, Maria Juliana Moura Corrêa, Marli Souza Rocha, Maryane Oliveira Campos, Olavo de Moura Fontoura, Sheila Maria Alvim de Matos, Walter Massa Ramalho

**Affiliations:** I Universidade Estadual de Feira de Santana, Núcleo de Epidemiología – Feira de Santana (BA), Brasil.; II Fundação Oswaldo Cruz, Escola Nacional de Salud Pública – Rio de Janeiro (RJ), Brasil.; III Universidade do Estado do Rio de Janeiro, Instituto de Medicina Social – Rio de Janeiro (RJ), Brasil.; IV Universidade do Estado da Bahia, Departamento de Ciencias de la Vida – Salvador (BA), Brasil; V Universidade Federal de Mato Grosso, Instituto de Salud Colectiva – Cuiabá (MT), Brasil.; VI Universidade Federal do Ceará, Facultad de Medicina – Fortaleza (CE), Brasil.; VII Universidade Federal de Minas Gerais, Facultad de Medicina – Belo Horizonte (MG), Brasil.; VIII Universidade Estadual de Campinas, Facultad de Ciencias Médicas – Campinas (SP), Brasil.; IX Universidade de São Paulo, Facultad de Medicina – São Paulo (SP), Brasil.; X Universidade Federal da Bahia, Instituto de Salud Colectiva – Salvador (BA), Brasil.; XI Universidade Federal do Rio de Janeiro, Instituto de Estudios en Salud Colectiva – Rio de Janeiro (RJ), Brasil.; XII Universidade Federal de Santa Catarina, Departamento de Salud Pública – Florianópolis (SC), Brasil.; XIII Ministerio de Salud, Secretaría de Vigilancia en Salud y Ambiente – Brasília (DF), Brasil.; Universidade Federal do Rio de Janeiro - Rio de Janeiro (RJ), Brasil.; Universidade Federal Fluminense - Niterói (RJ), Brasil.; Universidade de Brasília - Brasília (DF), Brasil.; Universidade do Estado do Rio de Janeiro - Rio de Janeiro (RJ), Brasil.; Universidade Federal de Pelotas - Pelotas (RS), Brasil.; Ministério da Saúde, Secretaria de Vigilância em Saúde e Ambiente - Brasília (DF), Brasil.; Organizacão Pan-Americana da Saúde - Brasília (DF), Brasil.; Organização Mundial da Saúde - Brasília (DF), Brasil.; Universidade de Brasília - Brasília (DF), Brasil.; Universidade Federal de Pernambuco - Vitória de Santo Antão (PE), Brasil.; Universidade do Estado do Rio de Janeiro - Rio de Janeiro (RJ), Brasil.; Universidade Federal da Bahia - Salvador (BA), Brasil.; Ministério da Saúde, Secretaria de Vigilância em Saúde e Ambiente - Brasília (DF), Brasil.; Universidade Federal do Rio Grande do Sul - Porto Alegre (RS), Brasil.; Fundação Oswaldo Cruz - Belo Horizonte (MG), Brasil.; Universidade Federal dos Vales do Jequitinhonha e Mucuri - Diamantina (MG), Brasil.; Universidade Federal do Acre - Rio Branco (AC), Brasil.; Universidade Federal do Maranhão - São Luís (MA), Brasil.; Ministério da Saúde, Secretaria de Vigilância em Saúde e Ambiente - Brasília (DF), Brasil.; Universidade Federal Fluminense - Niterói (RJ), Brasil.; Fundação Oswaldo Cruz - Manaus (AM), Brasil.; Ministério da Saúde, Secretaria de Vigilância em Saúde e Ambiente - Brasília (DF), Brasil.; Fundação Oswaldo Cruz - Manaus (AM), Brasil.; Universidade Federal do Ceará - Fortaleza (CE), Brasil.; Faculdade de Ciências Médicas da Santa Casa de São Paulo - São Paulo (SP), Brasil.; Fundação Oswaldo Cruz - Recife (PE), Brasil.; Fundação Oswaldo Cruz - Rio de Janeiro (RJ), Brasil.; Universidade Estadual de Campinas - Campinas (SP), Brasil.; Universidade Federal da Bahia - Salvador (BA), Brasil.; Universidade Federal do Recôncavo da Bahia - Santo Antônio de Jesus (BA), Brasil.; Universidade de São Paulo - São Paulo (SP), Brasil.; Universidade Federal de Minas Gerais - Belo Horizonte (MG), Brasil.; Universidade Federal de São João Del-Rei - Divinópolis (MG), Brasil.; Universidade de Brasília - Brasília (DF), Brasil.; Ministério da Saúde, Secretaria de Vigilância em Saúde e Ambiente - Brasília (DF), Brasil.; Organização Pan-Americana da Saúde - Brasília (DF), Brasil.; Fundação Oswaldo Cruz - Rio de Janeiro (RJ), Brasil.; Secretaria de Estado da Saúde de São Paulo - São Paulo (SP), Brasil.; Fundação Oswaldo Cruz - Brasília (DF), Brasil.; Ministério da Saúde, Secretaria de Vigilância em Saúde e Ambiente - Brasília (DF), Brasil.; Universidade Federal do Espírito Santo - Vitória (ES), Brasil.; Ministério da Saúde, Secretaria de Vigilância em Saúde e Ambiente - Brasília (DF), Brasil.; Universidade Federal do Rio de Janeiro - Rio de Janeiro (RJ), Brasil.; Universidade Estadual do Rio de Janeiro - Rio de Janeiro (RJ), Brasil.; Ministério da Saúde, Secretaria de Vigilância em Saúde e Ambiente - Brasília (DF), Brasil.; Universidade de Brasília - Brasília (DF), Brasil.; Ministério da Saúde, Secretaria de Vigilância em Saúde e Ambiente - Brasília (DF), Brasil.; Ministério da Saúde, Secretaria de Vigilância em Saúde e Ambiente - Brasília (DF), Brasil.; Ministério da Saúde, Secretaria de Vigilância em Saúde e Ambiente - Brasília (DF), Brasil.; Ministério da Saúde, Secretaria de Vigilância em Saúde e Ambiente - Brasília (DF), Brasil.; Ministério da Saúde, Secretaria de Vigilância em Saúde e Ambiente - Brasília (DF), Brasil.; Universidade Federal da Bahia - Salvador (BA), Brasil.; Universidade de Brasília - Brasília (DF), Brasil.

**Keywords:** Epidemiología, Planificación estratégica, Educación en salud, Investigación, Política de salud

## Abstract

La Asociación Brasileña de Salud Colectiva (Abrasco) ha sido protagonista en la consolidación de la salud colectiva en Brasil desde su fundación. Tras 19 años, la Comisión de Epidemiología de Abrasco retomó su planificación con la elaboración del V Plan Director para el Desarrollo de la Epidemiología en Brasil (2025–2029). Este plan surgió en un contexto desafiante, marcado por desigualdades sociales, negacionismo científico, pandemia de COVID-19, crisis climática y desinversión en políticas públicas, lo que refuerza la necesidad de actualización de la epidemiología brasileña. La elaboración del plan se llevó a cabo en múltiples etapas, incluyendo encuestas en línea, debates y talleres presenciales. El Taller I (Brasilia, 2023) identificó los desafíos prioritarios, y el Taller II (Río de Janeiro, 2024) definió estrategias de abordaje. El proceso de construcción se basó en la participación cualificada de epidemiólogos de todas las regiones del país y se fundamentó en principios de pluralidad, diversidad, equidad de género y raza/etnia. El plan destaca la epidemiología en tres áreas temáticas: formación; investigación; y políticas, programas y servicios de salud. El Grupo de Trabajo del Plan Director de la Comisión de Epidemiología, con el apoyo de la Secretaría de Vigilancia en Salud y Ambiente del Ministerio de Salud, condujo el proceso. Este suplemento de la *Revista Brasileira de Epidemiologia* presenta el V Plan Director para el Desarrollo de la Epidemiología en Brasil (2025–2029), contribuciones para debates sobre algunos temas centrales y profundización de dimensiones estratégicas y operativas. El V Plan Director para el Desarrollo de la Epidemiología en Brasil (2025–2029) expresa una visión colectiva de futuro orientada al fortalecimiento de la epidemiología como disciplina central en el campo de la salud colectiva y reafirma su compromiso con la equidad, la justicia social y la consolidación del Sistema Único de Salud en Brasil.

## PRESENTACIÓN

Hace 45 años surgió la *Associação Brasileira de Saúde Coletiva* (Abrasco) — denominada en ese momento Asociación Brasileña de Posgrado en Salud Colectiva. Constituida por destacados activistas de las áreas de salud pública y de medicina social y preventiva, la Abrasco se consolidó como un espacio central en la lucha por la reforma sanitaria, articulándose con movimientos sociales que demandaban la democratización del país^1^. En el período en que la población brasileña y la de otros países latinoamericanos se encontraba bajo regímenes cívico-militares, profesionales y trabajadores del ámbito de la salud en Brasil se incorporaban a los movimientos sociales por la democratización y desarrollaban acciones acompañadas de reflexiones críticas y análisis fundamentados en nuevos marcos teóricos. Estos análisis permitieron una comprensión renovada de la industria de la salud, de los sistemas sanitarios y de los determinantes de la salud de las poblaciones en sociedades capitalistas. De este modo, se conformó el campo de la salud colectiva, sostenido en perspectivas críticas y diferenciado de las concepciones predominantes en la salud pública tradicional y en la medicina preventiva^2^.

La creación de la Abrasco favoreció la articulación entre los participantes de esos movimientos científicos y de la lucha social, además de abrir espacios de diálogo — aun susceptibles de perfeccionamiento en la actualidad — entre los campos disciplinares de la epidemiología, las ciencias humanas y sociales en salud, la planificación, la gestión y las políticas de salud^3,4^. En este contexto de defensa de la justicia social y de fortalecimiento del Sistema Único de Salud (SUS), la epidemiología brasileña se consolidó como uno de los pilares de la salud colectiva. Desde entonces, su desarrollo ha transcurrido en constante interacción con los demás campos que integran el área — las ciencias sociales en salud y las políticas, planificación y gestión en salud —, incorporando los compromisos asumidos por la Abrasco: la construcción de una sociedad más equitativa y la promoción de mejores condiciones de vida y salud para toda la población brasileña^5^.

Desde sus inicios, la Abrasco, a través de su Comisión de Epidemiología, se ha organizado con el propósito de elaborar planes directores para el Desarrollo de la Epidemiología en Brasil. El primero de ellos fue formulado en 1989^6^ y el cuarto en 2005^7^. A lo largo del tiempo, estos documentos han establecido directrices y propuestas fundamentales que, una vez implementadas, contribuyeron de manera decisiva a la consolidación, expansión y cualificación de la epidemiología como campo disciplinar estratégico en el ámbito de la salud colectiva brasileña.

La elaboración de este V Plan Director para el Desarrollo de la Epidemiología en Brasil 2025-2029, 19 años después de la formulación del IV plan, se inscribe en un contexto en el que acontecimientos y transformaciones de gran impacto en Brasil y en el mundo configuran nuevos escenarios y generan diferentes desafíos^8^. En las últimas décadas, la expansión de las políticas neoliberales, en el marco de la financiarización del modo de producción capitalista, ha provocado concentración de la renta, incremento de la pobreza, precarización del trabajo, profundización de las inequidades sociales en salud y desfinanciamiento de las políticas de salud y de bienestar social. La pandemia de COVID-19, que tuvo lugar en Brasil bajo un gobierno negacionista, evidenció el avance de la difusión y de la adhesión de segmentos de la población a concepciones antivacunas y anticiencia, las cuales plantean nuevas cuestiones para el campo de la salud colectiva y, en particular, para la epidemiología. El escenario actual impone desafíos aún mayores para la defensa del SUS y la protección de la salud de la población brasileña. Entre los aspectos centrales que caracterizan el presente, sobresale como problema creciente la crisis climática. Asimismo, los importantes avances tecnológicos de los últimos años abren nuevas perspectivas de aplicación en el campo de la salud, aunque tales innovaciones deben ser acompañadas por evaluaciones críticas que consideren todas las dimensiones potencialmente impactadas.

Las actividades previstas en los cuatro planes directores anteriores contribuyeron a que la epidemiología, destacando su pertinencia intrínseca al campo de la salud colectiva, experimentara un exitoso proceso de expansión reflejado en sus respectivos niveles de actuación: formación, investigación y articulación con las políticas y los servicios de salud.

En el ámbito de la formación, se destaca la notable expansión de los programas de posgrado en salud colectiva/salud pública, así como la creación y el fortalecimiento de los cursos de grado en salud colectiva. Estos programas se extendieron, aunque de manera desigual, por las distintas regiones del país, alcanzando incluso procesos de interiorización.

En el ámbito de la investigación, las líneas de estudio se diversificaron y los resultados se evidencian tanto en el significativo aumento de la producción bibliográfica publicada en revistas y libros nacionales e internacionales como en la producción técnico-tecnológica, con avances relevantes en métodos y técnicas.

La calidad de los trabajos publicados equivale a la de aquellos producidos a nivel internacional, con amplia cobertura de los problemas de salud de la población brasileña. A modo de ejemplo, sin desmerecer otras contribuciones, puede mencionarse la producción de sólidos análisis epidemiológicos sobre la pandemia de COVID-19. Resulta fundamental destacar el papel de las agencias de fomento — *Conselho Nacional de Desenvolvimento Científico e Tecnológico, Coordenação de Aperfeiçoamento de Pessoal de Nível Superior* y *Fundações de Apoio à Pesquisa* —, que, por sus características, se han constituido, en la medida de su adecuada gestión, en instancias imprescindibles para el progreso científico.

En la articulación con las políticas y los servicios de salud, la participación y la contribución efectiva de la comunidad epidemiológica, guiadas por las directrices de los planes directores, han sido significativas. En este marco, la definición de agendas estratégicas y el seguimiento de las estructuras institucionales en los diferentes niveles del SUS cuentan con la colaboración activa, continua y cercana de la Abrasco, cuya actuación no se restringe al ámbito disciplinar de la epidemiología. Asimismo, el apoyo a la producción de conocimientos generados en el contexto de los servicios ha recibido de esta asociación aportes sustanciales para su gestión.

A pesar de los avances significativos alcanzados con la implementación de los cuatro planes anteriores, respaldados por la Organización Panamericana de la Salud (OPS) y el Ministerio de Salud, los nuevos desafíos y problemas en el ámbito de la salud requieren una actualización en los tres niveles de actuación mencionados previamente. En un contexto de defensa irrestricta de la democracia, de la Política Nacional de Ciencia, Tecnología e Innovación y del SUS, así como en la búsqueda de la superación de las inequidades/desigualdades sociales en salud, la elaboración del V Plan Director para el Desarrollo de la Epidemiología en Brasil 2025-2029 constituye un marco fundamental destinado a contribuir a los avances en el país.

Este V Plan Director presenta un diagnóstico amplio, contextualizado y actualizado de los problemas de salud que se enfrentan. Recupera, como referencia inicial, la síntesis de los planes anteriores, en particular del IV Plan Director, considerando las tres áreas temáticas: formación, investigación en epidemiología y epidemiología en políticas, programas y servicios de salud. En cada una de estas áreas, se reconocen nuevas dimensiones de los problemas previamente identificados y se incorporan nuevos desafíos, alineándose con el objetivo principal de consolidar progresivamente el campo de la salud colectiva.

Para garantizar los principios de pluralidad, diversidad, inclusión y una dirección democrática en la elaboración del plan, el proceso contó con la participación activa de epidemiólogos de todas las regiones de Brasil. Los participantes representaron diferentes generaciones de investigadores, docentes y trabajadores del SUS. Asimismo, se procuró asegurar la equidad de género y de raza/etnia, fortaleciendo las perspectivas de construcción colectiva y democrática basadas en el compromiso de la epidemiología con la reducción de las desigualdades sociales en salud.

Se espera que este V Plan Director, respaldado de manera sustancial por el Departamento de Acciones Estratégicas en Vigilancia en Salud de la Secretaría de Vigilancia en Salud y Ambiente del Ministerio de Salud, estructure un conjunto productivo de acciones, fomente desarrollo teórico y metodológico significativo y, sobre todo, impulse avances efectivos en el fortalecimiento del SUS, así como en la construcción de estrategias exitosas y participativas para la reducción de las desigualdades sociales en salud.

## V PLAN DIRECTOR PARA EL DESARROLLO DE LA EPIDEMIOLOGÍA EN BRASIL (2025–2029)

Después de 19 años, la Comisión de Epidemiología de la Abrasco retoma el proceso de construcción de otro Plan Director: el V Plan Director para el Desarrollo de la Epidemiología en Brasil, quinquenio (2025–2029).

El proceso de elaboración de este plan se estructuró en diferentes etapas. La primera consistió en la síntesis de los debates sobre la identificación de problemas y desafíos para la epidemiología, realizados en el I Taller del V Plan Director para el Desarrollo de la Epidemiología en Brasil, en Brasilia (DF), los días 17 y 18 de agosto de 2023. En la segunda etapa, se sistematizó una encuesta en línea dirigida a la comunidad de epidemiólogos que participó en el I Taller, con el objetivo de enumerar estrategias para abordar los problemas identificados. La tercera etapa, desarrollada durante el II Taller, realizado en Río de Janeiro (RJ) los días 12 y 13 de septiembre de 2024, permitió sistematizar todas las contribuciones previas. La información sobre las actividades y el perfil de los participantes en la elaboración del V Plan Director para el Desarrollo de la Epidemiología en Brasil 2025-2029 se encuentra en el Anexo 1.

A lo largo de todo el período, se llevaron a cabo diversas reuniones, tanto remotas como presenciales, con un intenso trabajo participativo orientado al logro de los resultados presentados.

La elaboración del V Plan Director para el Desarrollo de la Epidemiología en Brasil 2025-2029 fue posible gracias al trabajo dedicado y comprometido de numerosos epidemiólogos brasileños. Cabe destacar la labor del Grupo de Trabajo del Plan Director de la Comisión de Epidemiología de la Abrasco, que, a partir de 2022, planificó y condujo el proceso de elaboración de este plan. Asimismo, se reconoce la participación activa del equipo de la Secretaría de Vigilancia en Salud y Ambiente del Ministerio de Salud en dicho proceso. El producto aquí presentado constituye una síntesis de las múltiples contribuciones y de los debates desarrollados a lo largo de estos años y, al igual que los planes anteriores, refleja una visión de futuro para el desarrollo de la epidemiología brasileña.

## ÁREAS TEMÁTICAS: PROBLEMAS IDENTIFICADOS Y ACCIONES PROPUESTAS

### Área temática I: Formación en Epidemiología

#### Eje 1: Enseñanza de grado y posgrado

Problemas sistematizados:

- Desigualdades y asimetrías regionales en la distribución y asignación de recursos financieros para los programas de posgrado, tanto en la modalidad académica como en la profesional, en el país;
- Necesidad de fortalecer el intercambio y la comunicación entre los programas de grado y posgrado en el campo de la salud colectiva, con énfasis en la epidemiología, en todo el país;
- Los cursos y programas de formación presentan un desequilibrio entre la teoría y el análisis de la realidad epidemiológica, la cual continúa sin ser suficientemente explorada. Esta brecha ha limitado la capacidad de los estudiantes para desarrollar una comprensión crítica de los desafíos contemporáneos, dificultando su habilidad para aplicar teorías y conceptos en contextos específicos;
- Disociación entre la epidemiología y otros núcleos disciplinares que conforman las bases del campo de la salud colectiva, lo que repercute negativamente en el proceso de enseñanza-aprendizaje;
- Incorporación incipiente del conocimiento epidemiológico en el abordaje de contenidos relacionados con las desigualdades sociales en salud, así como en temas de educación ambiental, educación en derechos humanos, relaciones étnico-raciales y cuestiones relativas a las culturas afrobrasileña e indígena en los cursos de grado y posgrado;
- Incipiente incorporación del conocimiento epidemiológico en las prácticas clínicas de las distintas áreas de las ciencias de la salud;
- La formación en epidemiología, tanto en los niveles de grado como de posgrado, con frecuencia no logra responder de manera adecuada a las necesidades locales y regionales.
- Intensa fragmentación de la formación de posgrado, derivada de la estructuración de las áreas de concentración, sin la creación de canales permanentes de diálogo e intercambio de conocimientos, lo que genera barreras significativas para la interlocución entre los tres núcleos disciplinares de la salud colectiva, comprometiendo la formación integral deseada y necesaria para los epidemiólogos del país;
- Las estrategias pedagógicas no consideran de manera adecuada las habilidades ni las competencias específicas del perfil de los ingresantes y egresados, ni las diferentes modalidades de aprendizaje ni los objetivos particulares de cada proceso educativo.

Acciones recomendadas para el abordaje de los problemas identificados:

- Estimular la apertura estratégica y regionalizada de licenciaturas en salud colectiva en el país, en consonancia con las Directrices Curriculares Nacionales, que contemplen la enseñanza de la epidemiología en el nivel de grado, articulada con los programas de posgrado;
- Estimular la apertura de programas de posgrado en salud colectiva en regiones y áreas deficitarias, mediante financiamiento específico orientado a la reducción de desigualdades y asimetrías;
- Fortalecer las acciones de solidaridad y cooperación entre programas de posgrado consolidados y no consolidados, mediante la promoción de diferentes estrategias, como posdoctorados específicos para docentes de programas aún no consolidados;
- Fomentar la integración entre programas de posgrado, con el fin de promover el intercambio de experiencias en los procesos formativos. Este esfuerzo debe contemplar la identificación de prácticas exitosas, el análisis de las limitaciones enfrentadas y la discusión de los desafíos pendientes, con el objetivo de mejorar la formación académica y profesional;
- Incentivar el desarrollo de estrategias de atracción y retención de docentes, con el fin de consolidar los programas de posgrado y los grupos de investigación en regiones y áreas deficitarias;
- Crear canales que favorezcan la cooperación internacional por medio de redes colaborativas, destacando la importancia de las alianzas entre países del Sur Global para promover el intercambio de conocimientos, recursos e innovación, con énfasis en el desarrollo humano y social;
- Estudiar formas de implementar estrategias para la reanudación del financiamiento de iniciativas de cooperación entre instituciones, integrándolas al sistema de evaluación de los programas académicos. Asimismo, es fundamental establecer mecanismos sólidos de monitoreo y evaluación continua de estas iniciativas, garantizando su sostenibilidad y su impacto positivo en el desarrollo de colaboraciones interinstitucionales;
- Garantizar una formación en epidemiología alineada con las demás formaciones discursivas en el campo de la salud colectiva (ciencias humanas y sociales; política, planificación y gestión en salud), promoviendo una visión amplia e integrada. Esta perspectiva formativa busca reducir la fragmentación del conocimiento y permitir que los profesionales desarrollen una capacidad sólida para enfrentar problemas complejos en salud pública;
- Ampliar la oferta de programas de formación con foco en la construcción de modelos teóricos y en la comprensión de las dinámicas involucradas en los procesos de salud-enfermedad, que orienten desde la planificación de los instrumentos de investigación hasta el análisis crítico de los datos;
- Estimular la oferta de componentes curriculares con enfoque fundamentado en los referentes de la epidemiología social;
- Estimular la creación de componentes curriculares de enseñanza de la epidemiología en cursos de grado y posgrado, orientados a la preparación, vigilancia y respuesta a eventos que puedan constituir emergencias de salud pública de relevancia nacional. El enfoque se dirige a la formación de profesionales de la salud que participarán en la respuesta nacional frente a futuros desastres y pandemias;
- Incentivar la realización de diagnósticos situacionales sobre los componentes curriculares de epidemiología en los cursos de grado y posgrado en salud colectiva, evaluando la existencia de estrategias que promuevan la integración de este componente con otros que abordan los demás núcleos disciplinares del campo de la salud colectiva (ciencias humanas y sociales; política, planificación y gestión en salud);
- Incentivar la actualización de la matriz curricular de los cursos de formación en las distintas perspectivas formativas en epidemiología, incorporando una mayor carga horaria práctica, por ejemplo, mediante simulaciones basadas en la realidad;
- Estimular la creación de espacios de diálogo permanentes en la Abrasco para debates sobre la formación en epidemiología, un proceso fundamental para ampliar e intensificar el intercambio entre diferentes estrategias formativas y satisfacer así las necesidades del país;
- Apoyar la inclusión de la epidemiología en la curricularización de la extensión como iniciativa fundamental, dentro de un proceso desarrollado en estrecha colaboración entre las instituciones de educación superior y los movimientos sociales o comunitarios;
- Crear espacios regulares de diálogo entre docentes y estudiantes, tanto en los niveles de grado como de posgrado, para la evaluación continua de la formación en epidemiología dentro del campo de la salud colectiva, la identificación de lagunas e insuficiencias y la propuesta de soluciones para superarlas.

#### Eje 2: Formación Docente

Problemas sistematizados:

- La formación docente en estrategias pedagógicas y en tecnologías digitales para la enseñanza de la epidemiología es insuficiente, lo que provoca una preparación inadecuada para la aplicación de métodos de enseñanza innovadores y la integración de herramientas digitales que enriquezcan el proceso de aprendizaje. Esta limitación genera dependencia de enfoques tradicionales que, con frecuencia, no involucran a los estudiantes y restringen tanto la comprensión de los conceptos epidemiológicos como su aplicación práctica;
- La limitada formación en tecnologías digitales dificulta la adaptación a las demandas contemporáneas de la enseñanza, restringiendo la preparación de futuras generaciones de epidemiólogos y comprometiendo su capacidad para enfrentar los desafíos actuales en salud pública;
- Ausencia de iniciativas a nivel nacional e integradas, enfocadas en la formación docente en epidemiología.

Acciones recomendadas para el abordaje de los problemas identificados:

- Promover estudios sobre la docencia y sus aspectos didácticos para la evolución de las prácticas pedagógicas, lo que implica la evaluación continua de los métodos empleados, así como la discusión de nuevos enfoques y estrategias activas que puedan enriquecer el proceso de enseñanza-aprendizaje en epidemiología;
- Promover un mayor intercambio de experiencias entre los distintos centros de formación en epidemiología del país;
- Apoyar la formulación, discusión y difusión de métodos y estrategias pedagógicas activas para la enseñanza de la epidemiología, utilizando una mayor variedad de recursos tecnológicos y alineándose con las competencias y objetivos específicos de cada proceso de aprendizaje;
- Promover la integración de metodologías activas como estrategias pedagógicas para la enseñanza de la epidemiología en los componentes curriculares, asegurando que los procesos educativos sean inclusivos, motivadores y transformadores;
- Inducir la oferta de programas de formación para docentes, centrados en el uso de tecnologías digitales de información y comunicación, como parte de las políticas institucionales;
- Incentivar estrategias, como mesas redondas y talleres de trabajo, centradas en prácticas docentes específicas para la enseñanza de la epidemiología en el nivel de grado.

#### Eje 3: Articulación e Integración Enseñanza-Servicio-Investigación-Comunidad

Problemas sistematizados:

- Deficiencia en la formación integrada a la práctica en los servicios de salud, lo que evidencia desconexión entre la teoría impartida en los cursos y la realidad profesional;
- Limitación de los currículos, que no consideran las particularidades de cada área de formación ni la necesidad de un enfoque transversal entre las diferentes modalidades de enseñanza de la epidemiología;
- Respuesta limitada ante las demandas de los servicios de salud de la red de atención del SUS en la formación en epidemiología, tanto en los cursos de grado como en los programas de posgrado en las modalidades académica y profesional, particularmente respecto a las acciones de vigilancia;
- Deficiencia en la articulación y la interlocución entre las instituciones de enseñanza y los servicios de salud, lo que compromete la conexión con la realidad práctica y con los profesionales de la salud en el proceso formativo y de educación permanente;
- Insuficiencia de reflexión teórica y metodológica en el análisis de datos de los Sistemas de Información en Salud, así como en el uso de tecnologías digitales de información y comunicación, constituyendo un desafío significativo para la efectividad de las intervenciones en salud pública. Esta laguna ha limitado la elaboración de análisis críticos y fundamentados, reduciendo la capacidad de los profesionales de la salud para generar información relevante que sustente la toma de decisiones y la planificación estratégica;
- Uso limitado de *software* libre en la enseñanza de la epidemiología, en todos los niveles de formación, constituyendo una barrera significativa para la modernización y la mayor efectividad de la educación en esta área;
- Necesidad de una incorporación criteriosa y crítica de la inteligencia artificial (IA) en el sector de la salud. Si bien la IA tiene el potencial de transformar la atención a la salud, la gestión de datos y la investigación en este campo, su implementación debe acompañarse de un análisis cuidadoso de los impactos éticos, sociales y operativos;
- Avance incipiente en la incorporación de tecnologías relacionadas con la salud digital en las instituciones formadoras, en contraste con la creciente demanda de estas tecnologías en los servicios de salud, la vigilancia y los centros de referencia;
- Deficiencia en la capacidad de traducir los conocimientos (popularización y difusión) generados en los distintos procesos formativos en epidemiología, tanto para públicos específicos como para la sociedad en general.

Acciones recomendadas para el abordaje de los problemas identificados:

- Promover la inclusión de un componente curricular de vigilancia en salud en los cursos de grado en salud en general y en los de posgrado en salud colectiva, con un enfoque en fomentar el interés y el conocimiento de los estudiantes sobre prácticas eficaces para enfrentar los problemas de salud en el país;
- Estimular la creación de espacios de práctica en los servicios de salud, especialmente en el área de vigilancia en salud, dentro de los cursos de grado y posgrado en el campo de la salud colectiva;
- Promover pasantías extracurriculares en los cursos de grado que faciliten la aproximación de los estudiantes a la práctica de la epidemiología en los servicios de salud del SUS y en los territorios comunitarios;
- Promover iniciativas que favorezcan la aproximación y la integración de gestores(as) con profesionales de la salud en los procesos formativos y de educación permanente;
- Incorporar las necesidades de los servicios de salud en el desarrollo de la producción bibliográfica y técnico-tecnológica de las distintas modalidades de cursos, contribuyendo a que la formación académica responda a las demandas reales del SUS;
- Estimular estrategias de educación permanente en epidemiología, dirigidas a profesionales de la salud;
- Promover espacios de reflexión teórica y metodológica para el análisis de los Sistemas de Información en Salud y para la utilización de tecnologías digitales de información y comunicación;
- Fomentar la creación de un portal de acceso público que permita la disponibilidad de *software* libre enfocado en epidemiología;
- Estimular la formación de estudiantes, gestores e investigadores con habilidades para la aplicación de métodos y técnicas de traducción de los conocimientos (popularización y difusión) generados en la sociedad.

### Área temática II: La Investigación en Epidemiología

#### Eje 1: Desigualdades Sociales e Inequidades en Salud

Problemas sistematizados:

- Insuficiencia de investigaciones que integren el modelo económico en sus enfoques teóricos, en las propuestas metodológicas y en la evaluación de impactos sobre la salud;
- Número reducido de estudios y escasa producción de conocimiento sobre poblaciones en situación vulnerable, como personas viviendo en la calle, en privación de libertad, pueblos indígenas, poblaciones rurales/ribereñas, poblaciones del campo, de la selva y de las aguas, población LGBTQIAPN+, *quilombolas* y personas con discapacidad.

Acciones recomendadas para el abordaje de los problemas identificados:

- Reforzar la importancia de que las investigaciones enfaticen los orígenes y la producción de las desigualdades socioeconómicas generadas en el corazón del sistema capitalista, las cuales constituyen fuertes determinantes de inequidades en salud. Considerando el estado actual del proceso de concentración de la renta y de implementación de políticas neoliberales, se destaca la necesidad de ampliar el número de estudios que permitan monitorear el impacto de estos procesos sobre la pobreza, los indicadores de salud y la magnitud de las desigualdades sociales en salud;
- Además del impacto de este modelo económico, se recomienda que las condiciones de vida y de salud de la mayoría de la población brasileña, desfavorecida, excluida y oprimida, constituyan un foco prioritario de investigación. Dentro de estas poblaciones, se debe dar especial énfasis a la población negra y femenina, históricamente discriminada, desvalorizada y excluida;
- Estimular componentes curriculares críticos y discusiones periódicas sobre los impactos en la salud de las políticas económicas y sociales, del proceso productivo y de las desigualdades estructurales, de manera que se promueva la realización de investigaciones basadas en modelos teóricos sólidos que integren los determinantes sociales de la salud y las perspectivas de los distintos núcleos disciplinares de la salud colectiva;
- Fomentar el desarrollo de indicadores específicos para la medición de la interseccionalidad y la complejidad, integrando la transdisciplinariedad y una pluralidad de enfoques metodológicos, frente a los desafíos operativos de la incorporación de las desigualdades e inequidades en salud en los modelos de análisis;
- Incentivar investigaciones que promuevan la reflexión crítica sobre las diferentes teorías sociológicas aplicadas a los modelos de determinación social del proceso salud-enfermedad y de las condiciones de salud;
- Promover el uso plural, crítico y creativo de métodos de investigación en estudios sobre desigualdades sociales en salud, incorporando la participación de las comunidades estudiadas en el proceso investigativo, la adopción de indicadores adecuados para medir eventos de salud en poblaciones vulnerabilizadas y la consideración de la interseccionalidad. Esto puede incluir la formación de alianzas con organizaciones comunitarias, la realización de consultas previas y el respeto a las culturas y a los conocimientos tradicionales;
- Incentivar políticas públicas que promuevan la realización de estudios — como convocatorias, becas de investigación, entre otras — y la divulgación de resultados de investigaciones con enfoque en las desigualdades sociales en salud en Brasil. Esto incluye las desigualdades regionales, con énfasis en el impacto de las políticas macroestructurales y del proceso de concentración de la renta sobre la salud y la calidad de vida de las poblaciones vulnerabilizadas;
- Ampliar la articulación entre grupos de investigación y programas de posgrado para estudios colaborativos sobre inequidades en salud, promoviendo el intercambio de conocimientos, iniciativas, instrumentos y técnicas que diversifiquen y potencien los estudios en el área. Las desigualdades inter- e intrarregionales del país deben considerarse en estas iniciativas, por ejemplo, mediante la inclusión de eventos científicos con discusiones periódicas sobre el tema;
- Demandar el desarrollo de políticas públicas e iniciativas académicas, como la apertura de convocatorias de financiación específicas para el desarrollo de investigaciones estratégicas con distintas poblaciones en contextos de mayor vulnerabilidad;
- Ampliar la diversidad en los consejos editoriales y promover la proposición de números temáticos sobre subgrupos poblacionales en situación de mayor vulnerabilidad en revistas científicas del campo de la salud colectiva;
- Estimular la oferta de asignaturas y la realización de seminarios que incentiven la investigación sobre subgrupos poblacionales en situación de mayor vulnerabilidad;
- Incentivar y apoyar metodologías de investigación participativa que involucren a las comunidades estudiadas en sus territorios, mediante alianzas con organizaciones comunitarias y la realización de consultas previas, fortaleciendo el respeto a las culturas y a los conocimientos tradicionales;
- Impulsar cursos y talleres de trabajo especializados en investigación con grupos poblacionales en situación vulnerable en los congresos de epidemiología, promoviendo además una mayor diversidad e integración entre los grupos temáticos de la Abrasco que aborden temas relacionados;
- Reforzar e incentivar que las investigaciones relacionadas con poblaciones en situación de mayor vulnerabilidad se fundamenten en los derechos humanos y sociales, haciendo especial énfasis en las personas con discapacidad, particularmente aquellas que enfrentan una doble carga de discriminación, ya que, además de ver restringidos sus derechos fundamentales, son consideradas por el modelo económico neoliberal menos importantes para el sistema capitalista por ser supuestamente menos productivas.

#### Eje 2: Financiamiento para Investigaciones e Instituciones de Investigación

Problema sistematizado:

- Insuficiencia de políticas de financiamiento científico robustas y estables, lo que compromete la democratización, el fortalecimiento, la expansión y la internacionalización de los grupos de investigación brasileños.

Acciones recomendadas para el abordaje del problema identificado:

- Debatir con agencias de financiamiento y organismos gubernamentales municipales, estatales y federales la necesidad de establecer políticas de financiamiento más robustas y estables para las investigaciones epidemiológicas, así como fortalecer las iniciativas de apoyo mediante la colaboración entre países del Sur Global;
- Estimular el fortalecimiento de políticas de financiamiento que apoyen a jóvenes doctores, previniendo la deserción en la carrera científica y la fuga de talentos al exterior. Esto incluye la disponibilidad de becas de fijación o de posdoctorado para la participación en redes colaborativas, así como la apertura de concursos públicos para la incorporación de personal en universidades, institutos y fundaciones, acompañados de la infraestructura adecuada para garantizar las condiciones necesarias para la conformación de grupos de investigación;
- Movilizar a la comunidad científica para generar evidencia sobre el impacto de la inversión pública en ciencia y tecnología en el desarrollo del país, en el avance del conocimiento y en la mejora de las políticas públicas, de manera que se pueda sostener las demandas ante las agencias de financiamiento y organismos gubernamentales por recursos de investigación continuos y robustos;
- Estimular que las agencias de financiamiento consideren las desigualdades regionales al abrir convocatorias de investigación, actuando con equidad y atendiendo a las necesidades específicas de cada región del país y de las poblaciones más vulnerables;
- Ofrecer oportunidades de formación para que los jóvenes investigadores puedan elaborar propuestas competitivas y gestionar los recursos de manera eficaz, ampliando la capacidad de captación de fondos nacionales e internacionales para el fomento de la investigación epidemiológica en el país;
- Exigir que la selección de proyectos y grupos de investigación financiados con recursos públicos se realice prioritariamente mediante convocatorias públicas ampliamente difundidas, restringiendo las contrataciones directas a situaciones específicas, como aquellas relacionadas con emergencias en salud pública;
- Promover la discusión sobre financiamiento para repositorios de datos confiables que cumplan con la Ley General de Protección de Datos Personales y que estén disponibles para el uso de investigadores y gestores de la salud.

#### Eje 3: Teoría, Método y Ética en Investigación

Problemas sistematizados:

- Brecha teórica y limitaciones metodológicas en las investigaciones epidemiológicas, lo que restringe el desarrollo de estudios con impactos sociales significativos;
- Insuficiencia de enfoques multidimensionales e interdisciplinarios que permitan abordar la complejidad de los problemas y contextos de la salud;
- Uso de datos secundarios en salud con poca reflexión y profundización sobre sus potencialidades y limitaciones para el análisis epidemiológico;
- Acceso limitado a bases de datos gubernamentales con fines de investigación;
- Falta de repositorios confiables y seguros para el almacenamiento de datos derivados de investigaciones epidemiológicas, con miras a la implementación de estrategias de ciencia abierta;
- Inexistencia de debate sobre la adecuación de los aspectos éticos en las investigaciones poblacionales.

Acciones recomendadas para el abordaje de los problemas identificados:

- Valorar la formación crítica de los investigadores, fortaleciendo en ellos la necesidad de desarrollar estudios con una sólida reflexión teórica y la búsqueda de métodos de investigación que permitan avances significativos en el conocimiento y generen evidencia sólida para abordar problemas concretos de salud;
- Estimular la creación y el fortalecimiento de redes de investigación para la difusión de teorías y métodos, incorporando programas de posgrado y grupos de investigación de distintas regiones y niveles de desarrollo;
- Desarrollar acciones de formación continua para docentes e investigadores, promoviendo el fortalecimiento e innovación en los métodos y teorías de la investigación epidemiológica;
- Incentivar el debate y la implementación de iniciativas que reduzcan la valoración del productivismo científico, promoviendo el apoyo a investigaciones con relevancia científica y social y con profundización teórica y metodológica y la adopción de buenas prácticas editoriales y de publicación científica;
- Estimular una mayor integración entre grupos de investigación y programas de posgrado de distintas áreas y vocaciones, así como promover el desarrollo de convocatorias específicas que exijan o incentiven la formación de equipos de investigación con experiencia diversa, incluyendo disciplinas como sociología, antropología, psicología, salud pública, economía y estudios culturales;
- Formar grupos de trabajo para identificar estrategias aplicables a la salud colectiva y a la investigación epidemiológica alineadas con la estrategia de salud digital para Brasil que estimulen la investigación, la innovación y el desarrollo de soluciones en IA, con énfasis en el desarrollo de soluciones para problemas relevantes para la salud de la población brasileña;
- Estimular el financiamiento de estudios orientados al desarrollo de herramientas para la investigación epidemiológica y a la evaluación de su implementación en los servicios de salud;
- Incentivar la cooperación internacional para el intercambio de nuevas tecnologías, buenas prácticas y experiencias exitosas en salud colectiva desarrolladas en otros contextos nacionales;
- Promover eventos y cursos que capaciten a los investigadores en el uso cualificado y creativo de los datos secundarios de salud. Incentivar que los cursos de grado, posgrado y técnicos aborden la importancia del análisis de datos de los Sistemas de Información en Salud y orienten estudios que favorezcan avances en el conocimiento y su aplicación por parte de los servicios;
- Apoyar la educación permanente de los equipos de vigilancia y de otros profesionales de la salud en la recolección e ingreso de datos en los Sistemas de Información, con el objetivo de mejorar su calidad, completitud y uso cualificado por parte de los servicios de salud;
- Promover la colaboración entre profesionales de epidemiología, estadística, ciencias humanas y sociales, gestión de datos y otros colectivos, de manera que el análisis de datos secundarios se realice con múltiples perspectivas y utilizando diversos métodos y técnicas;
- Exigir la mejora continua de las bases de datos existentes en Brasil, especialmente en lo relativo a la ampliación de cobertura, actualización, cualificación de la información y estandarización de variables, con el fin de facilitar la integración entre los Sistemas de Información del Ministerio de Salud y de otros sectores gubernamentales;
- Dialogar con el Ministerio de Salud y otros organismos gubernamentales sobre la necesidad de garantizar acceso abierto, democrático y transparente a los datos secundarios de interés epidemiológico en toda su extensión, respetando la Ley General de Protección de Datos Personales;
- Crear espacios de diálogo entre investigadores, representantes del control social y operadores y gestores de bases de datos, con el fin de interpretar de manera adecuada y uniforme la Ley General de Protección de Datos Personales, garantizando el uso ético de los datos y, al mismo tiempo, facilitando su acceso;
- Promover debates sobre financiamiento y desarrollo de repositorios nacionales de datos confiables y seguros, que cumplan con la Ley General de Protección de Datos Personales y estén disponibles para el uso de investigadores y gestores de la salud;
- Desarrollar materiales instructivos y fomentar debates más abiertos con investigadores y gestores sobre la importancia de la ciencia abierta, la gestión segura de datos y el uso adecuado de repositorios;
- Fomentar debates sobre iniciativas innovadoras para mejorar las normas y los flujos de procedimientos éticos, adaptándolos a los distintos tipos de estudios poblacionales observacionales en epidemiología.

### Área Temática III: La Epidemiología en Políticas, Programas y Servicios de Salud

#### Eje 1: Sistemas Nacionales de Información y Prácticas Epidemiológicas: Análisis de Situación de Salud según las Condiciones de Vida

Problemas sistematizados:

- Limitación de la interoperabilidad entre los múltiples Sistemas de Información en Salud y tecnologías utilizadas en el SUS, así como con los Sistemas de Información de otros sectores o instituciones gubernamentales para análisis epidemiológicos. Estas dificultades se manifiestan en la fragmentación operacional, las restricciones técnicas y/o semánticas, y la inexistencia de un identificador único estandarizado para los ciudadanos, lo que reduce las posibilidades de acceso y la intercomunicación en el uso efectivo de los datos disponibles;
- Insuficiencia en la disponibilidad y calidad de los datos, así como en el acceso y uso de los Sistemas de Información en Salud para cubrir las dimensiones epidemiológicas de enfermedades/afecciones/eventos y sus determinantes, considerando especialmente a las poblaciones más vulnerables en los distintos contextos sociales del país;
- Restricción en el acceso a datos de la vigilancia en salud y de las Redes de Atención a la Salud, incluyendo información proveniente de planes y servicios de salud privados, como clínicas, hospitales, laboratorios, farmacias y unidades de vacunación;
- Fragilidad en las acciones de consolidación de datos en el SUS, así como en los análisis epidemiológicos y la divulgación de información, lo que limita la retroalimentación oportuna y cualificada de datos críticos para la toma de decisiones y su difusión a la sociedad;
- Insuficiencia de inversiones para garantizar la infraestructura y el personal cualificado necesarios para la gestión en salud, con énfasis en el mantenimiento, actualización y operacionalización de los Sistemas de Información en Salud en los distintos niveles de gestión del SUS.

Acciones recomendadas para el abordaje de los problemas identificados:

- Fortalecer, en el ámbito de Abrasco, la estructuración de espacios estratégicos que amplíen la participación técnico-científica en instancias de toma de decisiones en salud en el Estado brasileño, legitimados mediante el reconocimiento de grupos de trabajo temáticos ya existentes o que se constituyan en la Comisión de Epidemiología;
- Abogar para que la propiedad y la gestión de los datos provenientes de los Sistemas Nacionales de Información en Salud sean responsabilidad exclusiva del sector público del país, alojados en centros de datos dentro del territorio nacional e indelegables, y basados en principios éticos, democráticos y soberanos;
- Defender un plan de inversiones en el país para la consolidación de acciones estratégicas para el acceso a la información en salud, así como su disponibilidad y calidad, a nivel federal y en estados y municipios, mediante la actuación de espacios legítimos para la toma de decisiones y con criterios para la aplicación responsable de la Ley General de Protección de Datos Personales ante las prioridades del país;
- Proponer que los Sistemas Nacionales de Información consideren la creación de un código identificador único para cada ciudadano(a) y la estandarización del perfil identificador del ciudadano — un conjunto mínimo de datos (inserción cualificada de campos de nombre social, identidad de género, orientación sexual, raza, etnia, personas con discapacidad, entre otros) —, salvaguardando los principios de la Ley General de Protección de Datos Personales;
- Defender el acceso oportuno a datos del sistema privado de salud, considerando la diversidad de instituciones, servicios y planes de salud, con un enfoque principal en las acciones de vigilancia, así como en investigaciones estratégicas en salud para el país, incluyendo contextos de emergencias en salud pública;
- Indicar acciones que contribuyan a la sostenibilidad de los Sistemas Nacionales de Información mediante la Red Nacional de Datos en Salud, garantizando la interoperabilidad técnica, operacional y semántica de las codificaciones, de manera integrada y disponible para otros sectores, como educación, ámbitos sociales y medio ambiente;
- Contribuir a la ampliación de la producción de conocimiento técnico-científico, atendiendo a la necesaria equidad frente a las desigualdades regionales y a las condiciones socialmente determinadas, con énfasis en las poblaciones y territorios más vulnerables, fundamentado en la consolidación y el análisis de bases de datos de los Sistemas Nacionales de Información y en sus interfaces intersectoriales;
- Recomendar la adopción y el perfeccionamiento de directrices y políticas estratégicas para el acceso a datos de salud, basadas en el principio fundamental del derecho a la información, tanto para datos provenientes de la vigilancia en salud como de las Redes de Atención a la Salud del SUS.

#### Eje 2: Formación de Personas

Problemas sistematizados:

- Desafíos en la formación de grado y posgrado para ampliar la comprensión y el uso de la epidemiología en el abordaje de la infodemia, las informaciones falsas, el negacionismo y la desinformación, con base en una visión crítica de la evidencia generada por los estudios;
- Desafíos para fomentar procesos formativos más inclusivos, que contemplen la diversidad humana, incluyendo orientación sexual, identidad de género, raza, etnia, clase social y personas con discapacidad;
- Inexistencia de carreras y contratos estables para profesionales de la salud pública con formación en epidemiología, que respondan a las necesidades de las políticas públicas del SUS para la toma de decisiones;
- Insuficiencia de inversiones en la Política Nacional de Educación Permanente en Salud (PNEPS) para el fomento de iniciativas que promuevan el análisis epidemiológico, la evaluación de impacto, el uso de informes de situación de salud, la comunicación en salud y la explotación completa del potencial de las bases de datos disponibles.

Acciones recomendadas para el abordaje de los problemas identificados:

- Potenciar las estrategias de formación, educación permanente, monitoreo, evaluación, supervisión, información y comunicación en el SUS, con un enfoque en la cualificación de la gestión y en la adopción de prácticas epidemiológicas de calidad. Estas estrategias deben apoyar la construcción de indicadores relevantes y fomentar el análisis consistente de la situación de salud para la toma de decisiones, así como cualificar la formación de los profesionales de la salud en cursos técnicos, de grado y de posgrado;
- Ampliar las acciones de promoción y la constitución de comités técnico-científicos para la implementación de la carrera de salubrista en el SUS, conforme a las normativas legales vigentes del Congreso Nacional, con una sólida base formativa en epidemiología. Estas acciones deben aplicarse en las redes de atención y vigilancia, fortaleciendo la gestión en salud y considerando competencias, responsabilidades y criterios específicos para cada nivel en las acciones de gestión, atención, vigilancia, prevención, control, investigación y docencia;
- Contribuir a la implementación de la PNEPS mediante iniciativas que fomenten la incorporación de nuevas estrategias basadas en el razonamiento y el método epidemiológico con enfoque territorial en las acciones de vigilancia, con el fin de ampliar la capacidad de ejecución de análisis epidemiológicos prioritarios. Esto incluye análisis situacionales, monitoreo y evaluación de procesos, así como la evaluación de efectividad, costo-beneficio e impacto de las intervenciones en salud;
- Contribuir a la adopción de prácticas formativas en los cursos técnicos de salud (nivel medio), de grado y de posgrado (*lato sensu*, con énfasis en residencias, y *stricto sensu*, con énfasis en cursos de maestría y doctorado en la modalidad profesional), así como en la educación permanente de trabajadores(as) de la salud, que consideren la diversidad humana, incluyendo orientación sexual, identidad de género, raza, etnia, clase social y personas con discapacidad, mediante políticas afirmativas y otras estrategias orientadas a superar las persistentes desigualdades sociales en el país;
- Proponer el fortalecimiento de estrategias formativas innovadoras que integren conocimientos sobre nuevas herramientas para la sistematización y el análisis de datos, así como la traducción del conocimiento técnico-científico (popularización y diseminación) en bases sólidas de información y comunicación en salud en el país, con el objetivo de ampliar la capacidad nacional de respuesta frente a situaciones de infodemia, divulgación de informaciones falsas, negacionismo y desinformación.

#### Eje 3: Vigilancia en Salud

Problemas sistematizados:

- Insuficiencia de articulación/integración entre la atención y la vigilancia en salud en el SUS, considerando todos los puntos e instancias de las Redes de Atención a la Salud según los ciclos de vida, las enfermedades, afecciones, eventos y determinantes sociales;
- Limitación de la efectividad de los componentes de la vigilancia en salud para las poblaciones más vulnerables, incluyendo pueblos indígenas, *quilombolas*, asentadas, personas viviendo en la calle, migrantes, refugiadas y apátridas, privadas de libertad, personas LGBTQIAPN+, así como comunidades gitanas y circenses;
- Insuficiencia en la incorporación de estrategias innovadoras de vigilancia, incluyendo vigilancia popular y participativa, vigilancia genómica, identificación oportuna de nuevos patógenos/variantes, vigilancia predictiva y uso de redes sociales para la detección de eventos;
- Limitación en la incorporación de múltiples dimensiones territoriales y de contextos locales, lo que aumenta los desafíos asociados al abordaje de los determinantes sociales y los factores de riesgo;
- Limitación en la disponibilidad de información simplificada para la gestión en salud y para la sociedad, lo que dificulta la traducción del conocimiento (popularización y diseminación) y obstaculiza una comunicación y participación plena en salud.

Acciones recomendadas para el abordaje de los problemas identificados:

- Estimular la implementación de investigaciones prioritarias en las diversas realidades del SUS, promoviendo una participación social activa y un enfoque en la cualificación de las acciones de vigilancia en salud con interfaces en las Redes de Atención a la Salud, valorando perspectivas transdisciplinares e interepistémicas para abordar las desigualdades sociales y las inequidades en salud;
- Contribuir al desarrollo y a la amplia difusión de indicadores estratégicos de la vigilancia en salud de base territorial en sus distintas dimensiones, incluyendo aquellos multicriterios que integren perspectivas de determinantes sociales, para la definición de áreas prioritarias, con enfoque en el diseño y la implementación de acciones orientadas al enfrentamiento de los diversos desafíos y problemas reconocidos en el SUS;
- Contribuir a la adopción de evidencias generadas por los Sistemas Nacionales de Información para la toma de decisiones en el SUS, fundamentadas en métodos y técnicas epidemiológicas, e integradas a estrategias de base territorial, tales como la vigilancia popular, ciudadana y participativa, la vigilancia genómica, la identificación oportuna de nuevos patógenos o mutantes, la vigilancia predictiva, el uso de redes sociales para la detección de rumores y eventos, así como la incorporación de nuevas tecnologías;
- Cooperar en la implementación de métodos y técnicas epidemiológicas para el análisis de desigualdades sociales e inequidades en el SUS, así como fomentar la articulación transdisciplinaria, interepistémica e intersectorial necesaria en las iniciativas gubernamentales que aborden esta temática desde la perspectiva de la salud colectiva;
- Establecer prioridades para la cualificación, estructuración y organización de las acciones de la red nacional de laboratorios de salud pública, como parte integral de la vigilancia en salud de base territorial;
- Defender la ampliación del financiamiento de políticas y programas que integren acciones estratégicas del complejo económico e industrial de la salud, con el fin de superar las barreras de acceso a la salud y garantizar su carácter de derecho humano en el país;
- Defender la implementación efectiva de la Política Nacional de Vigilancia en Salud y de la Política Nacional de Salud del Trabajador y la Trabajadora, asegurando su integración con la Política Nacional de Atención Primaria a través de agendas de trabajo conjuntas y de una operacionalización intersectorial.

#### Eje 4: Evaluación de Programas, Servicios e Intervenciones en Salud

Problemas sistematizados:

- Fortalecer las iniciativas de monitoreo y evaluación epidemiológica del impacto de los modelos de vigilancia en salud, considerando los contextos actuales del país, de manera que sirvan como fuente confiable de evidencia para su reformulación efectiva;
- Fortalecer las políticas, programas y acciones integradas para la estructuración y organización de la red de laboratorios (tanto públicos como privados), de manera que respalden eficazmente la estructuración, el monitoreo y la evaluación de las acciones de vigilancia en salud;
- Insuficiencia en la financiación para el desarrollo de acciones de monitoreo y evaluación epidemiológica;
- Limitación del proceso de inducción para que los programas de posgrado profesionales (maestrías y doctorados) incorporen el desarrollo de estudios epidemiológicos con temáticas prioritarias para el país en la implementación, el monitoreo y la evaluación de programas, servicios e intervenciones en salud;
- Limitación del alcance de las perspectivas aportadas por la epidemiología para responder a las demandas y prioridades nacionales de manera articulada con las acciones del Ministerio de Salud.

Acciones recomendadas para el abordaje de los problemas identificados:

- Ampliar y fortalecer en el país los canales de divulgación de la producción bibliográfica en epidemiología, como revistas, eventos técnico-científicos, sitios *web*, plataformas y repositorios, para difundir acciones exitosas desarrolladas en diferentes territorios y espacios de gestión del SUS, priorizando perspectivas fundamentadas en la determinación social y el diálogo con la sociedad brasileña;
- Avanzar en el desarrollo, cualificación, valoración y divulgación de producciones técnicas y tecnológicas vinculadas a análisis situacionales, con el objetivo de proponer soluciones a problemas inherentes a la vigilancia y a la atención, incluyendo estrategias formativas en los cursos técnicos de salud (nivel medio), de grado y de posgrado (*lato sensu*, con énfasis en residencias, y *stricto sensu*, especialmente en los cursos de maestría y doctorado en la modalidad profesional), así como en la educación permanente de trabajadores(as) en los territorios y servicios del SUS;
- Proponer el desarrollo de iniciativas para la cualificación de las acciones de monitoreo y evaluación, con enfoque en un sistema descentralizado e integrado de datos e información de la vigilancia y de las Redes de Atención a la Salud. Dichas iniciativas deben valorar la consolidación por su construcción democrática, respetar las diversidades regionales y poblacionales, y reconocer experiencias exitosas, como las presentadas en la Muestra Nacional de Experiencias Exitosas en Epidemiología, Prevención y Control de Enfermedades;
- Defender la ampliación de la infraestructura de tecnología de la información en la red de atención y vigilancia del SUS, con la adecuación de equipos y la mejora de la cobertura y calidad de internet, para la implementación de flujos operacionales cualificados y seguros, contando con personal debidamente capacitado;
- Defender la ampliación del financiamiento destinado a acciones formativas estratégicas que fortalezcan la capacidad de planificación, monitoreo y evaluación basadas en el método epidemiológico, con énfasis en la cualificación de la toma de decisiones en los distintos niveles de gestión del SUS, promoviendo la participación de instituciones de enseñanza e investigación en la formación de profesionales mediante estrategias innovadoras, como el Programa de Educación por el Trabajo para la Salud para la vigilancia en salud y los cursos de posgrado en la modalidad profesional;
- Contribuir al desarrollo de métodos y técnicas de monitoreo y evaluación participativa de las acciones de producción de información y de incorporación de nuevas tecnologías en todos los niveles de atención, vigilancia y gestión del SUS.

#### Eje 5: Inserción de la Epidemiología en las Políticas Intra e Intersectoriales

Problemas sistematizados:

- Dificultad de implementación de las perspectivas y enfoques de la epidemiología sobre las inequidades en salud, así como de la articulación inter- y transdisciplinaria entre las tres formaciones discursivas del campo de la salud colectiva;
- Heterogeneidad y considerable complejidad en el uso de la epidemiología en los distintos niveles de gestión del SUS y entre ellos, para la toma de decisiones basada en evidencia en políticas públicas;
- Insuficiente valorización y divulgación de las acciones exitosas de la epidemiología llevadas a cabo en territorios y espacios de gestión del SUS;
- Insuficiencia de iniciativas de colaboración en red entre programas de posgrado (en salud colectiva), investigadores(as), instituciones y servicios de salud, orientadas a fortalecer la integración extensión-enseñanza-servicio-comunidad como política de Estado, y no únicamente de gobierno.

Acciones recomendadas para el abordaje de los problemas identificados:

- Contribuir a la ampliación de la movilización y el compromiso social, con amplia participación de la sociedad civil y garantizando representatividad y diversidad, mediante liderazgos, movimientos y organizaciones sociales, en los espacios sociopolíticos de decisión de la Abrasco, potenciando acciones estratégicas exitosas de la epidemiología desarrolladas en distintos territorios y espacios de gestión del SUS;
- Contribuir a la construcción de espacios de mayor integración entre los comités temáticos y los grupos de trabajo de Abrasco, con el objetivo de reconocer y fortalecer las iniciativas estratégicas de formación y comunicación en red de cursos de grado y programas de posgrado, promoviendo la solidaridad y la colaboración. Estas iniciativas deben integrar, traducir y divulgar las prioridades de salud de la sociedad brasileña, alcanzadas mediante acciones de vigilancia en salud, incluyendo prácticas epidemiológicas. Asimismo, deben favorecer la ampliación de la integración extensión-enseñanza-servicio-comunidad y valorar espacios democráticos, participativos y legítimos en el país;
- Contribuir a la ampliación de estrategias de movilización y participación de liderazgos, representantes de Consejos de Salud y movimientos sociales por la educación, con el fin de fortalecer la Red Nacional de Formación en Vigilancia en Salud del SUS, promoviendo su carácter ciudadano, democrático y participativo.

## Figures and Tables

**Figura 1:** Número de participantes de los talleres según institución de origen.

Fiocruz: Fundação Oswaldo Cruz; OPS: Organización PanAmericana de la Salud; SVSA: Secretaria de Vigilância em Saúde e Ambiente; UFAC: Universidade Federal do Acre; UFBA: Universidade Federal da Bahia; UFC: Universidade Federal do Ceará; UFPE: Universidade Federal de Pernambuco; UFMA: Universidade Federal do Maranhão; UFRB: Universidade Federal do Recôncavo Baiano; UEFS: Universidade Estadual de Feira de Santana; UNB: Universidade de Brasília; UFMT: Universidade Federal do Mato Grosso; UERJ: Universidade do Estado do Rio de Janeiro; UFRJ: Universidade Federal do Rio de Janeiro; UFF: Universidade Federal Fluminense; USP: Universidade de São Paulo; Unicamp: Universidade Estadual de Campinas; UFVJM: Universidade Federal dos Vales do Jequitinhonha e Mucuri; UFSJ: Universidade Federal de São João del-Rei; UFSC: Universidade Federal de Santa Catarina; UFRGS: Universidade Federal do Rio Grande do Sul; UFPEl: Universidade Federal de Pelotas.

## Data Availability

Todo el conjunto de datos que respalda los resultados de este estudio está disponible bajo solicitud a la autora correspondiente, Tânia Maria de Araújo. El conjunto de datos no está públicamente disponible debido a la presencia de información sensible con potencial de identificación de los/las participantes. La recolección de estos datos se realizó en contextos colaborativos y requiere un manejo ético y responsable. Por lo tanto, el acceso es restringido para garantizar la privacidad de los involucrados y respetar los principios de protección de datos personales, en cumplimiento de las exigencias éticas relacionadas con la confidencialidad de los participantes.

## ANEXO 1.

### Información adicional sobre el proceso de elaboración del V Plan Director para el Desarrollo de la Epidemiología en Brasil (2025–2029) y el perfil de los(las) participantes involucrados(as).

El proceso de elaboración del V Plan Director para el Desarrollo de la Epidemiología en Brasil (2025–2029) se estructuró en dos fases:

- Fase 1: Diagnóstico de los principales problemas evaluados en tres áreas temáticas: I. Formación; II. Investigación; III. La Epidemiología en Políticas, Programas y Servicios de Salud;
- Fase 2: Propuesta de acciones y estrategias para abordar los problemas prioritarios definidos en la fase 1.

En cada fase se realizó una encuesta electrónica, se elaboraron informes técnicos preliminares y se promovió un taller presencial para discusión y deliberación. La primera encuesta tuvo como objetivo identificar los problemas prioritarios en cada eje, sirviendo de base para los informes que orientaron el primer taller.

En Brasilia, los días 17 y 18 de agosto de 2023, se llevó a cabo el **I Taller** para la elaboración del **V Plan Director para el Desarrollo de la Epidemiología en Brasil (2025–2029)**, ejecutando así la **fase 1** del proceso de elaboración. El diagnóstico de los problemas se consolidó en un informe técnico, derivado de las discusiones y la relatoría de los grupos en las tres áreas temáticas mencionadas anteriormente. En la Figura 1 se describen las características generales del grupo participante de los talleres.

Con base en la información del informe técnico de la **fase 1** (definición de los problemas prioritarios), se diseñó una encuesta para recopilar sugerencias de acciones para superar los problemas identificados. Así, se envió a los/las participantes del I Taller un formulario electrónico para registrar sugerencias de estrategias de abordaje de los problemas.

Como preparación para el II Taller, se elaboró un informe técnico preliminar con las sugerencias recogidas mediante la encuesta electrónica. El informe recopiló contribuciones destinadas a orientar las discusiones y definir acciones para el desarrollo de la epidemiología en los cinco años siguientes, las cuales fueron discutidas y aprobadas en el II Taller.

El II Taller se realizó los días 12 y 13 de septiembre de 2024, en Río de Janeiro, con el objetivo de ejecutar la **Fase 2**, enfocada en la discusión y definición de las propuestas de acción.

El proceso de elaboración del Plan Director involucró un amplio conjunto de epidemiólogos de todas las regiones del país. La Tabla 1 presenta el perfil de los/las participantes involucrados/as en al menos una de las etapas realizadas, incluidos los cuestionarios electrónicos y los talleres.

**Tabla 1:** Perfil de los/las participantes en las diferentes etapas del proceso de elaboración del V Plan Director para el Desarrollo de la Epidemiología en Brasil (2025–2029). Brasil, 2023–2024.

Características		%
Región		
	Norte	4,5
	Nordeste	20,9
	Centro-Oeste	18,2
	Sudeste	40,9
	Sur	15,5
**Características generales de los/las informantes**		
Género		
	Mujer cisgénero	60,7
	Hombre cisgénero	36,4
	Travesti	1,0
	Prefiero no informar	1,9
Rango etario (años)*		
	Hasta 41	26,7
	42–50	22,9
	51–64	25,7
	65 o más	24,8
Raza/color		
	Blanca	77,5
	Negra y parda	21,5
	Amarilla (asiática)	1,0
Grado académico**		
	Medicina	28,7
	Nutrición	13,9
	Odontología	7,4
	Enfermería	6,5
	Fisioterapia	6,5
	Farmacia	5,6
	Educación Física	4,6
	Estadística	4,6
Lugar de trabajo principal		
	Universidad pública	66,6
	Gestión pública em salud	21,2
	Centro de investigación	9,1
	Universidad privada	3,1
Tiempo trabajando como epidemiólogo/a (años)		
	Hasta 10	25,7
	11–15	19,0
	16–25	26,7
	26 o más	28,6
Campo de actuación actual***		
	Actividad académica/enseñanza	78,8
	Actividad de investigación	84,8
	Actividad en servicios de salud	36,4

* Media de edad: 51,2 años; mediana: 51,0 años; edad mínima: 29 años; edad máxima: 79 años;

** Otras graduaciones referidas: Salud Colectiva (n=3; 2,8%), Biomedicina (n=3; 2,8%), Ciencias Biológicas (n=3; 2,8%), Fonoaudiología (n=3; 2,8%), Ciencias Sociales (n=1; 1,9%), Ingeniería (n=1; 1,9%), Geografía (n=1; 1,9%), Matemática (n=1; 1,9%), Psicología (n=1; 1,9%), Trabajo Social (n=1; 1,9%);

*** Variable con múltiples respuestas.
